# Predicting stereotactic radiosurgery outcomes with multi-observer qualitative appearance labelling versus MRI radiomics

**DOI:** 10.1038/s41598-023-47702-8

**Published:** 2023-11-28

**Authors:** David A. DeVries, Terence Tang, Ali Albweady, Andrew Leung, Joanna Laba, Carol Johnson, Frank Lagerwaard, Jaap Zindler, George Hajdok, Aaron D. Ward

**Affiliations:** 1 Department of Medical Biophysics, Western University, London, N6A 3K7 Canada; 2 Gerald C. Baines Centre, London Health Sciences Centre, London, N6A 5W9 Canada; 3 Department of Radiation Oncology, London Health Sciences Centre, London, N6A 5W9 Canada; 4 Department of Radiology, Unaizah College of Medicine and Medical Sciences, Qassim University, 56219 Buraidah, Saudi Arabia; 5 Department of Medical Imaging, Western University, London, N6A 3K7 Canada; 6 Department of Oncology, Western University, London, N6A 3K7 Canada; 7 Department of Radiation Oncology, Amsterdam University Medical Centre, Amsterdam, 1081 The Netherlands; 8 Department of Radiation Oncology, Haaglanden Medical Centre, Den Hague, 2512VA The Netherlands; 9 Holland Proton Centre, Delft, 2629JA The Netherlands

**Keywords:** Cancer imaging, CNS cancer, Radiotherapy, Biomedical engineering, Software

## Abstract

Qualitative observer-based and quantitative radiomics-based analyses of T1w contrast-enhanced magnetic resonance imaging (T1w-CE MRI) have both been shown to predict the outcomes of brain metastasis (BM) stereotactic radiosurgery (SRS). Comparison of these methods and interpretation of radiomics-based machine learning (ML) models remains limited. To address this need, we collected a dataset of n = 123 BMs from 99 patients including 12 clinical features, 107 pre-treatment T1w-CE MRI radiomic features, and BM post-SRS progression scores. A previously published outcome model using SRS dose prescription and five-way BM qualitative appearance scoring was evaluated. We found high qualitative scoring interobserver variability across five observers that negatively impacted the model’s risk stratification. Radiomics-based ML models trained to replicate the qualitative scoring did so with high accuracy (bootstrap-corrected AUC = 0.84–0.94), but risk stratification using these replicated qualitative scores remained poor. Radiomics-based ML models trained to directly predict post-SRS progression offered enhanced risk stratification (Kaplan–Meier rank-sum *p* = 0.0003) compared to using qualitative appearance. The qualitative appearance scoring enabled interpretation of the progression radiomics-based ML model, with necrotic BMs and a subset of heterogeneous BMs predicted as being at high-risk of post-SRS progression, in agreement with current radiobiological understanding. Our study’s results show that while radiomics-based SRS outcome models out-perform qualitative appearance analysis, qualitative appearance still provides critical insight into ML model operation.

## Introduction

Brain metastases (BMs) form when cancer spreads to the brain, and are a hallmark of advanced disease. As improvements in cancer treatments have increased patients’ life expectancies, their risk of developing BMs at some point during the course of their disease has increased to 10–20%^1^. Given the symptoms and risks associated with BMs, and the short median survival of 8–16 months^2^ associated with BMs, it is critical that BM patients receive the most appropriate treatment as soon as possible.

Treatment options for BMs currently include surgical resection, whole brain radiation therapy (WBRT), or stereotactic radiosurgery (SRS)^3,4^. Surgical resection is recommended for patients with a favourable prognosis who present with few and accessible BMs. WBRT irradiates the entire brain and is non-invasive, avoiding standard surgical risk, but has a higher prevalence of neurocognitive decline post-treatment. In contrast, SRS limits the risk of neurocognitive toxicity by targeting only the BMs with radiation delivered with high doses in 1–3 fractions^5^. Stereotactic radiotherapy (SRT) is similar to SRS, except that it is delivered in more than three fractions, usually to larger BMs or post-surgical cavities.

SRS is highly effective, but up to 30% of treated BMs can progress post-SRS, constituting a treatment failure^5^. Escalation of SRS dose may reduce this failure rate, but may also increase the risk of toxicity^6,7^. Therefore, developing predictive models of BM response to SRS would aid in decision making to balance the risk of treatment failure and toxicity.

Previous studies have used qualitative interpretation of BM appearance in pre-treatment contrast-enhanced T1-weighted magnetic resonance imaging (T1w-CE MRI) or X-ray computed tomography to predict the outcome of WBRT^8^ and SRS^9–13^. These studies labelled BMs as either being “homogeneous”, “heterogeneous”, or “ring-enhancing”, and generally found that “homogeneous” BMs had the lowest risk of progression post-SRS, followed by “heterogeneous”, and then finally “ring-enhancing” BMs. It was hypothesized that the uniform uptake of contrast by a BM’s vasculature shown by “homogeneous” enhancement indicates strong oxygenation, which is linked to enhanced cell kill from radiation exposure.

**Figure 1:**
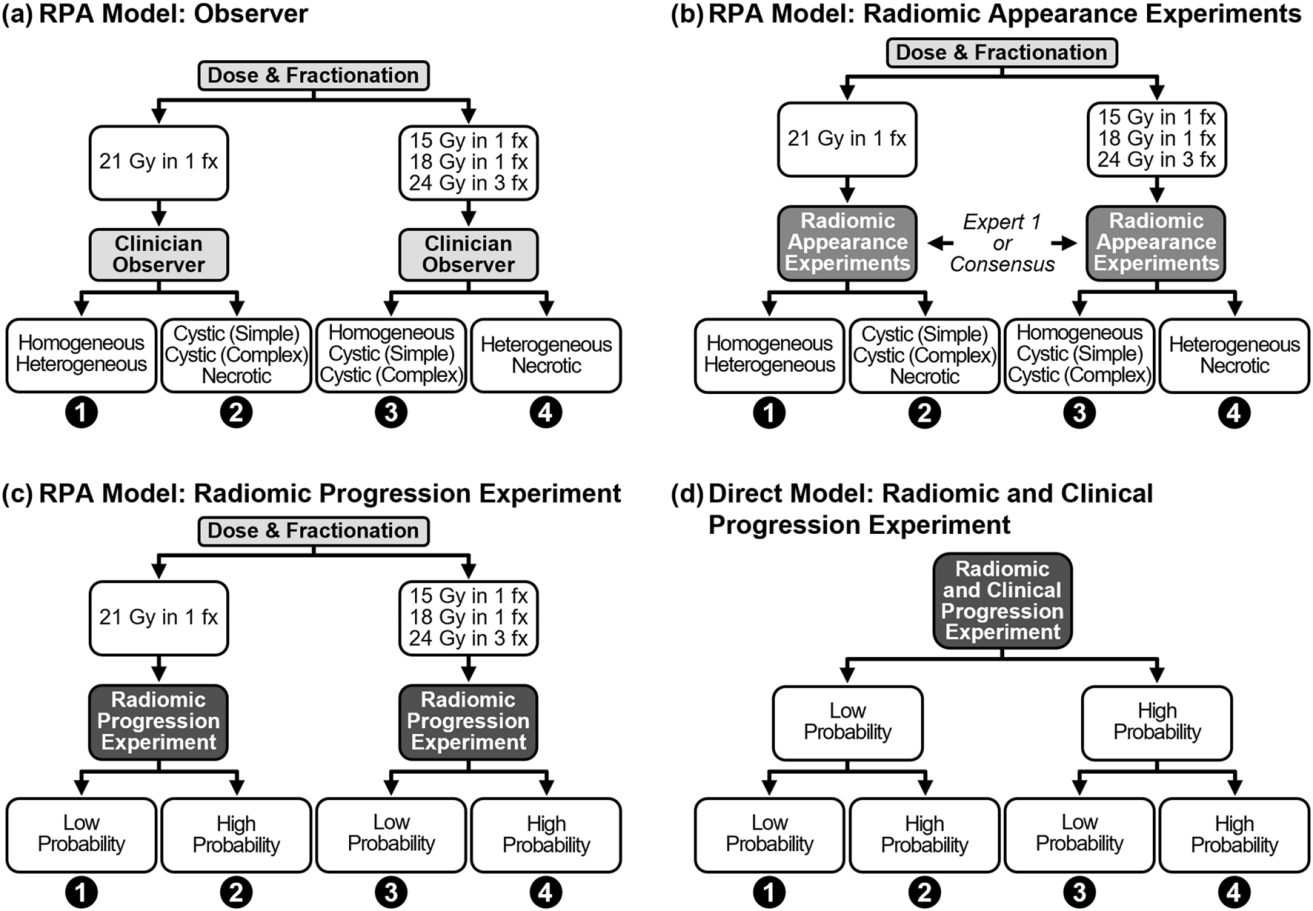
Models used for risk stratification of BMs for progression post-SRS. Each model stratifies BM into four risk groups, with group “1” intended to be BMs at the lowest risk for progression and each next group being at higher risk for progression. (**a**) Shows the original RPA model. The “Clinician Observer” could be any of the five observers, as well as the expert observer consensus. (**b**) Shows the replacement of the “Clinician Observer” in (**a**) with the five radiomic appearance label experiments’ results, each using one of the five appearance labels scored by either Expert 1 or the expert consensus. (**c**) Shows the integration of the radiomic progression experiment results into the RPA model structure. While the left and right branches use the same experimental results from a single ML experiment using the entire dataset, they use different ROC operating points to separate “low” versus “high” probabilities of progression. The direct model in (**d**) is no longer based on the RPA model, but rather only on the radiomic and clinical progression experiment’s results. Three ROC operating points (one at each split in the tree) were used to assign a risk group for a BM’s given probability of progression. Supplementary Fig. S2 provides further details on the ML experiments used for (**b**–**d**) and how ROC operating points were chosen for the probability splits in (**c**) and (**d**). Abbreviation: fx (fractions).

Rodrigues et al.^14^ presented a more advanced BM qualitative appearance interpretation using five (instead of three) qualitative labels: “homogeneous”, “heterogeneous”, “cystic (simple)”, “cystic (complex)”, and “necrotic”. In a recursive partitioning analysis (RPA) on multiple outcome predictors, they produced a model (henceforth referred to as “the RPA model”) that stratified BMs by risk of progression post-SRS using the SRS dose and fractionation prescription, and BM appearance (Fig. 1a). The BMs receiving a less aggressive SRS prescription were at a higher risk of progression, and within this group, BMs labelled as “heterogeneous” or “necrotic” were at the highest risk of progression. In contrast to the other appearance scoring approaches described above, this RPA model was specifically developed for outcome prediction using multiple variables and uses the most descriptive qualitative appearance labels to date, and so represents the most advanced predictive model of progression post-SRS using qualitative appearance scoring. While this RPA model has potential clinical benefit, the interobserver variability of the qualitative appearance labelling has not yet been measured.

More recently, machine learning (ML) and quantitative radiomic analysis of MRI have also been used to successfully predict outcomes of SRS^15–23^, post-surgical SRS^24^, and SRT^25–28^. Radiomics involves extracting quantitative features from medical images, which are then typically coupled with complex ML models to predict a value of interest^29,30^. While these radiomic-based ML models provide objective, quantitative, automated, and highly accurate prediction of SRS outcomes, their complexity and the abstract nature of their underlying radiomic features causes their operation to remain largely uninterpretable. ML models can be used clinically without complete interpretation of their operation, but this lack of model interpretation can make clinical translation and future research hypothesis generation more difficult.

Comparison between qualitative and quantitative approaches to BM SRS outcome prediction is also important. Kawahara et al.^18^ and Gutsche et al.^16^ both produced radiomic ML models predicting post-SRS progression, and compared them to a simple qualitative model using only the three-way “homogeneous”, “heterogeneous”, or “ring-enhancing” appearance labelling. Both found their ML models to be superior to the qualitative models, which were found to have low accuracy (44–62%). Neither study examined the more specific five-way appearance labelling of the RPA model, the more clinically relevant multi-variable RPA model using this labelling, nor performed risk stratification analysis to directly compare their results to the original BM qualitative appearance studies. Radiomic ML model operation could also be interpreted using qualitative appearance labels, allowing for model behaviour to be linked to biological hypotheses, encouraging clinical translation and informing research efforts.

Given the open questions surrounding the use and comparison of qualitative and quantitative MRI analysis for BM SRS outcome prediction, we conducted a study that uses established MRI radiomic techniques and presents novel analysis that examines the interobserver variability of qualitative appearance labelling, compares qualitative and quantitative techniques, and uses qualitative appearance labels to interpret complex radiomic models. Our study therefore addresses the following research questions:

1. What is the interobserver variability of the BM qualitative appearance scoring used by the RPA model?
2. Does the interobserver variability in appearance scoring decrease the RPA model’s ability to stratify BMs for risk of post-SRS progression?
3. Can radiomics-based ML models replicate the qualitative appearance labelling performed by clinicians, and what impact do these models’ inaccuracies have on risk stratification using the RPA model?
4. Do radiomics-based ML models directly predicting post-SRS progression provide enhanced risk stratification compared to qualitative appearance-based models?
5. Do BMs of different qualitative appearance labels have different probabilities of post-SRS progression predicted by radiomics-based ML models?
6. Are the feature importance scores of radiomics-based ML models correlated with biologically-relevant qualitative appearance labels?

## Methods

### Study sample

Our study’s sample consisted of 99 patients randomly selected from the original cohort studied by Rodrigues et al.^14^, for which the pre-treatment T1w-CE MRI was made available. The original cohort was collected retrospectively at the Amsterdam University Medical Centre (AUMC, The Netherlands), and so excluded patients with highly symptomatic BMs or poor prognosis. For the retrospective data collection, only patients who received first-line SRS for newly diagnosed, radiologically confirmed BMs were included. Any patients without pre-treatment or follow-up MRI were excluded. All patients were treated between 2003 and 2011 using linear-accelerator based SRS on a Novalis/Novalis TX unit (BrainLAB, Feldkirchen, Germany). BMs were prescribed 15, 18, or 21 Gy in one fraction, or 24 Gy in three fractions, with smaller BMs receiving the more aggressive prescriptions. Up to three BMs were treated concurrently per patient, and so a total of *n* = 123 BMs were individually analyzed.

### Clinical features and study endpoint

For each patient and BM, a set of 12 clinical features was available. These features were patient sex, age, primary cancer active status, primary cancer site, primary cancer histology, extracranial metastases status, systemic therapy status, neurological symptoms response to corticosteroids, and Eastern Cooperative Oncology Group (ECOG) score, along with per BM volume, location (supra vs. infratentorial), and SRS prescription. Supplementary Table S1 provides a summary of all the clinical features and their distributions for our study sample. Since Kaplan–Meier (KM) analysis was performed, post-SRS survival or follow-up length per patient was also collected to provide censorship data.

For each BM, post-SRS progression was defined radiographically using longitudinal post-treatment MRI. As the original dataset was collected before the introduction of the standardized Response Assessment in Neuro-Oncology Brain Metastases (RANO-BM) protocol^31^, a non-standard measurement technique was used. In this technique, each BM’s maximum diameters in three perpendicular directions (superior-inferior, mediolateral, posterior-anterior) were measured by a single, expert radiation oncologist and then their product was taken. If this product increased by ≥ 25% in any of the follow-up MRI post-SRS, the BM would be scored as “progression” (or positive/+) and scored as “no progression” (or negative/−) otherwise, defining a binary progression label. The RANO-BM protocol requires a maximum diameter across the BM without exiting the BM to be measured, and so RANO-BM scores could not be calculated from the existing progression scoring technique.

A confounder in all BM studies that use radiographically-defined endpoints is pseudo-progression, in which a BM can appear to grow in size, but this growth is unrelated to true cancerous progression^6^. All BMs scored as progression were reviewed by an expert clinician, who used longitudinal MRI and accompanying patient medical records to determine if pseudo-progression was present. If pseudo-progression had occurred, the BM was rescored as “no progression” (−).

### Imaging data and radiomic features

For each patient, pre-treatment T1w-CE MRI and BM region-of-interest (ROI) contours were collected. T1w-CE MRI was acquired pre-SRS for radiation treatment planning. Five scanner models were represented in our study sample, across which a total of eight different acquisition orientation and voxel size configurations were used (see Supplementary Table S2 for full details). Each BM’s ROI was defined by the gross tumour volume (GTV) planning contour that was manually drawn in three-dimensions by an expert clinician using the outer edge of the MRI contrast enhancement.

The MRI data was pre-processed and then radiomic features were extracted for each BM’s ROI. To account for variability in voxel resolution and intensity scaling across MR scanner models, the MRI data was pre-processed by first using the mean and standard deviation of all voxel values within the brain to apply a Z-score normalization at three standard deviations^32^. The image was then linearly interpolated to a common voxel size of 0.5 × 0.5 × 0.5 mm^3^, as 0.5 mm was the smallest voxel dimension present in the dataset (Supplementary Table S2). After pre-processing, 107 radiomic features were extracted from the MRI for each BM’s ROI using PyRadiomics v3.0.1^29^ in Python v3.6.13 with 64 intensity bins used where applicable (full feature list in Supplementary Table S3).

### Machine learning experimental design

Our ML experiments were all conducted using Matlab 2019b v9.7.0.1190202 (The Mathworks Inc., Natick, USA) based on a common template. This template consisted of a random decision forest (RDF) model that was trained and tested using a 250-iteration bootstrapped resampling technique across patients to avoid data leakage. During each iteration, the training dataset would be used to perform inter-feature correlation filtering, hyper-parameter optimization, and training of the RDF, which then would be tested using the iteration’s testing dataset (further details in Supplementary Figure S1 and Table S4).

For each ML experiment performed in our study, error metrics were calculated by aggregating the prediction probabilities of each testing dataset across all bootstrapped iterations. We used these probabilities to form an average receiver operating characteristic (ROC) curve with associated 95% confidence interval (CI) and area under the ROC (AUC). The average AUC from a bootstrapped resampling experiment is known to underestimate performance, and so we separately reported the common AUC_0.632+_ correction^33^. To calculate the misclassification rate (MCR), false negative rate (FNR), and false positive rate (FPR), an operating point on the ROC curve needed to be chosen without using the ROC curve itself to prevent bias. To do so, the predicted probabilities of the training dataset out-of-bag samples from the RDF training process were aggregated across bootstrapped iterations to form an ROC curve based on the training datasets. The upper-left operating point with the shortest distance to point (0, 1) on this ROC curve was found and then transferred to the testing datasets’ ROC curve, allowing the MCR, FPR, and FNR with associated 95% CIs to be calculated (see Supplementary Figure S1 for more details).

### Qualitative appearance interobserver variability

To investigate the question of interobserver variability in appearance labelling and replicability of the RPA model (research question 1), we recruited four clinicians from the London Health Sciences Centre (LHSC) in Canada (see Table 1) to repeat the labelling of each BM to compare against Rodrigues et al.’s original observer (henceforth referred to as “Expert 1”)^14^. This new set of clinicians relabelled all BMs as “homogeneous”, “heterogeneous”, “cystic (simple)”, “cystic (complex)”, or “necrotic” using only the same pre-treatment T1w-CE MRI available originally to Expert 1. We developed a custom application in Slicer v4.11.20210226^34^ that was used to sequentially display interactive axial, sagittal, and coronal views of each BM to the observers and record their appearance labelling results. After the appearance labelling, confusion matrices across the five appearance labels were created for each possible pair of observers, agreement rates were calculated (number of BMs with same appearance label from both observers divided by total number of BMs), and Fleiss’ kappa test was performed using SAS v9.4 (SAS Institute Inc., Cary, USA).

**Table 1:** Study observers that provided qualitative appearance labels for all BMs.

Observer	Clinical speciality	Medical centre	Initials
Expert 1	Radiation Oncology	VUMC, The Netherlands	J.Z
Expert 2	Radiation Oncology	LHSC, Canada	J.L
Expert 3	Neuro-radiology	LHSC, Canada	A.L
Trainee 1	Radiation Oncology	LHSC, Canada	T.T
Trainee 2	Neuro-radiology	LHSC, Canada	A.A

To address research question 2, we then applied the RPA model to each BM (Fig. 1a), but with the appearance labels from each observer. KM analysis of the BM risk of progression stratification was then performed per observer and SAS was used to perform the KM log-rank test across all groups. The appearance labels across the three expert observers were also used to generate a set of “expert consensus” qualitative labels, in which BMs received the common label chosen by a majority of the expert observers. Separate KM analysis was also performed using this expert consensus labelling with the RPA model.

### Qualitative appearance machine learning models

We explored research question 3 on replicating qualitative appearance labelling with ML by first using the BM qualitative appearance labels from Expert 1 to train ML models. As shown in Supplementary Fig. S2a, bootstrapped resampling ML experiments based on the previously described template were performed using the radiomics features as model input and one of the appearance labels as the model output. Therefore, an ML experiment was performed to label BMs as “homogeneous” or “not homogeneous”, another for labelling them as “heterogeneous or “not heterogeneous”, and similarly for the remaining appearance labels. The term “radiomic Expert 1 appearance experiments” is defined here to refer to these ML experiments. A binary model was trained for each of the five qualitative appearance labels. Binary models were used instead of a single five-way model as it allowed for the radiomic signature of each appearance label to be individually analyzed. This design decision therefore allows the model interpretation analysis described in a subsequent section to be performed for each qualitative appearance.”

The results of the five radiomic Expert 1 appearance experiments were first used to calculate error metrics associated with replicating each appearance label. For each experiment, the testing dataset prediction probabilities could be aggregated across the bootstrapped iterations to provide an average prediction probability per BM. The distance of this average probability per BM from each experiment’s optimal ROC operating point could then be found, and the maximum value taken across the five appearance label experiments to assign a qualitative appearance label to each BM (see Supplementary Fig. S2a). The RPA model was then used again, except with the appearance labels assigned by the ML models (as shown in Fig. 1b), and the same KM analysis performed. This entire methodology was then repeated in additional experiments to also explore using the expert consensus appearance labels to train the ML models instead of the appearance labels from Expert 1. These experiments will be referred to as the “radiomic consensus appearance experiments”.

### Post-SRS progression machine learning models

To address research question 4, we then examined the scenario in which instead of training ML models to provide qualitative appearance labels, the radiomic features would be used to directly predict if a BM would progress post-SRS. A ML model would therefore be unconstrained by trying to replicate clinician-based qualitative appearance labels and be able to find arbitrary radiomic signatures that were the most predictive of BM SRS response. This is the same technique employed by other ML studies, and so allowed our study to compare between the qualitative-based and quantitative-based approaches to SRS outcome prediction.

First, an ML experiment was conducted using only the radiomic features as the model input and BM post-SRS progression as the output using the common experiment template (see Supplementary Fig. S2c). This experiment will be henceforth referred to as the “radiomic progression experiment”. Error metrics were calculated from the experiment results along with average predicted probabilities of progression per BM across the bootstrapped iterations. As shown in Fig. 1c, the SRS dose and fractionation prescription split from the RPA model was still used to provide the first stratification of BMs, but after this the average predicted probability of progression from the ML experiment replaced the qualitative appearance labels to perform the final stratification splits. To provide comparison to the other RPA model results, the same KM analysis was performed on this new BM risk stratification.

Next, a second ML experiment was conducted using both the radiomic and clinical features to predict post-SRS progression, henceforth referred to as the “radiomic and clinical progression experiment” (see Supplementary Fig. S2d). This approach allowed for the RPA model to be completely unused, with the ML model instead being able to completely optimize the use of clinical and radiomic features to make its post-SRS progression predictions. To provide comparison to the RPA models results, a stratification with four risk groups was still performed, and so a two-layer splitting of BMs based on their average predicted progression probability from the ML experiment was used, as shown in Fig. 1d.

### Post-SRS progression machine learning model interpretation

To answer research question 5 and gain insight into the radiomic signature from the radiomic progression experiment, we first analyzed if the predicted probabilities from the radiomic progression experiment’s ML models were related to the more interpretable qualitative appearance labels. To do so, we took the average predicted probabilities of post-SRS progression per BM and then grouped them based on the expert consensus appearance labels (see Supplementary Fig. S2b). The statistical distributions of the predicted probabilities per appearance label were then compared using the Kruskal–Wallis test to compare distribution separation across all appearance labels.

We also analyzed the feature importance scores inherently provided by the RDFs trained within our experiments to investigate research question 6 by examining if any features were important in both the radiomic progression experiment and the radiomic consensus appearance experiments. For a given experiment, the RDF importance scores were normalized between 0 and 1 for each bootstrapped iteration, with 1 representing the most important feature. Features that were removed before model training by the inter-feature correlation filter received a score of 0. Each feature’s scores were then averaged across all bootstrapped iterations, and then renormalized between 0 and 1. We then selected the highly important features from the radiomic progression experiment (importance score ≥ 0.75) and determined whether these features were similarly important for any of the radiomic consensus appearance experiments.

Lastly, the identified highly important features underwent accumulated local effects (ALE) analysis. ALE analysis provides plots for each feature in which the change in a complex ML model’s predicted probability (of post-SRS progression or an appearance label) is shown on the y-axis as a function of the change in the feature’s value along the x-axis, spanning from the feature’s minimum to maximum value represented in the dataset. ALE plots therefore show if a feature leads to an increase or decrease in a model’s predicted probability (negative or positive ALE plots values), as well as at which values of the feature these changes in predicted probability occur. The calculation details for ALE plots are given in the following references^35,36^, which we applied by calculating a feature’s ALE values for each bootstrapped iteration in an experiment, and then averaging across iterations to get a single ALE plot per feature. This calculation was performed for each highly important feature across the six radiomic progression and consensus appearance experiments. For a single feature, the ALE plots of post-SRS progression and a given qualitative appearance were compared by taking the Pearson correlation coefficient (*ρ*) between them. If a strong positive correlation coefficient was found, then the feature predicted both progression and the qualitative appearance for the same values of the feature.

### Ethics declaration

The collection and analysis of the retrospective patient data used in this study was approved by the AUMC Medical Ethical Review Committee and was conducted within the approved guidelines. As the study was retrospective on a cohort of deceased patients, written consent from study participants was waived by the AUMC Medical Ethical Review Committee.

## Results

### Qualitative appearance interobserver variability

**Figure 2:**
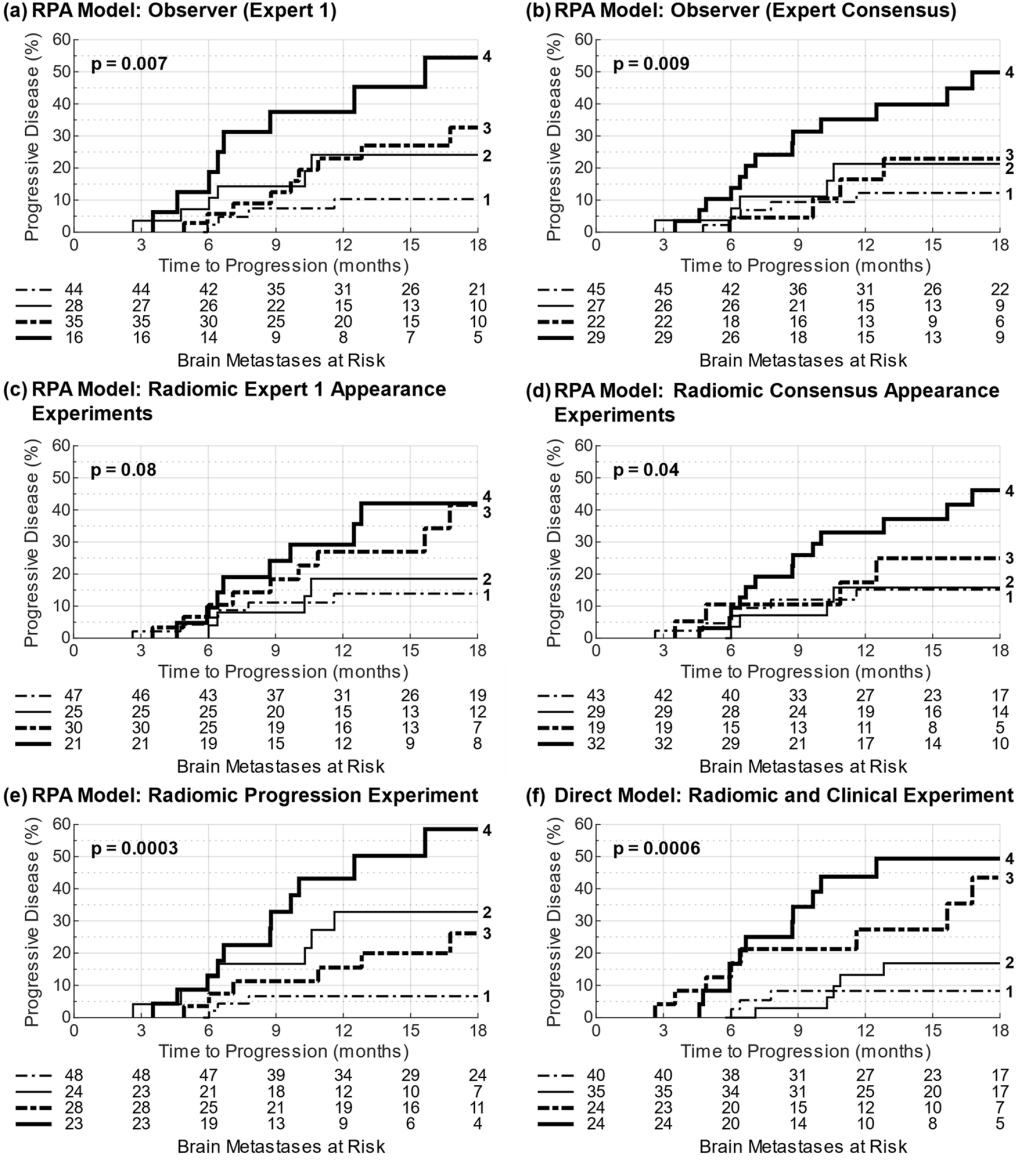
KM analysis for risk of a BM progressing post-SRS for the six primary risk stratification models evaluated (**a**–**f**). The risk group number for each risk curve is labelled on the right y-axis, and the number of BMs at risk per 3-month follow-up interval is given below each x-axis. The stated *p*-values are from the log-rank test performed over all risk groups.

Using the 123 BM sub-sample from the original Rodrigues et al. study^14^, we re-established the baseline performance of the RPA model using the BM appearance labels from Expert 1 as shown in Fig. 2a’s KM analysis for progressive disease. As would be expected, the KM results closely matched those of the original study and demonstrated statistically significant risk stratification, though at a lower level of significance due to the reduced number of samples. This first result therefore acted as a validated baseline of risk stratification performance against which to compare further results.

When observers relabelled the BM qualitative appearances, agreement with Expert 1’s labels ranged between 32.5 and 50.4% (see Table 2). The relabelling observers’ results were also compared to each other, with Expert 2, Expert 3, and Trainee 2 showing higher agreement rates between 61.8 and 65.9% (Table 2). Across all observers, Fleiss’ kappa test confirmed low agreement at κ = 0.38, with κ = 0.33 across only expert observers. The labelling confusion matrices showed that across all observers, 32.5% of disagreements involved “heterogeneous” labels, 23.8% included “necrotic” labels, and the remaining labels were each involved in 14.1–15.0% of disagreements (all data in Supplementary Table S5). Similarly, when considering only the expert observers, the disagreement rates were 30.9% “heterogeneous”, 23.6% “necrotic”, and 13.6–16.2% for each of the other labels. Analysis of the most common disagreements across all observers was also performed (Supplementary Table S6a). This analysis revealed that the highest rates of disagreement were between heterogenous and necrotic appearances (25.3% of disagreements), followed by mislabelling between heterogeneous and homogeneous appearances (21.4%). The results were similar across only the expert observers (Supplementary Table S6b), with 24.6% of disagreements between heterogeneous and homogeneous appearances, and 24.1% between heterogeneous and necrotic appearances.

**Table 2:**
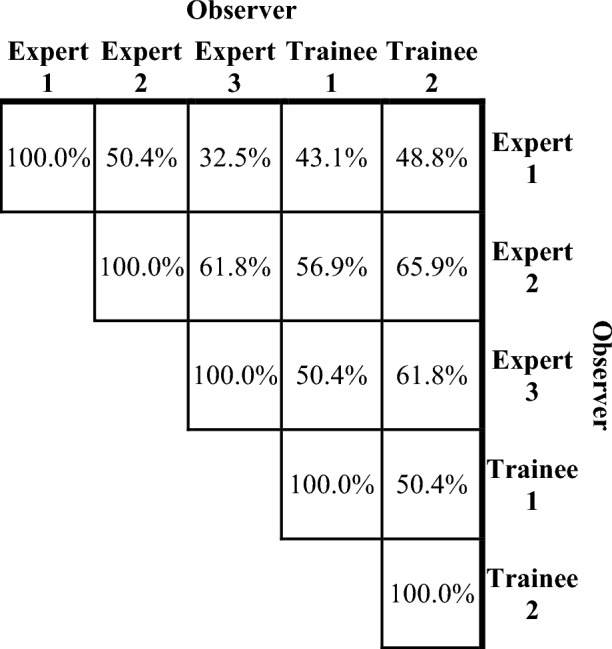
Qualitative appearance labelling agreement rates between all pairs of observers.

We found the labelling disagreement between observers impacted the RPA model risk stratification, with the additional expert observers (Experts 2 and 3) having KM log-rank test *p*-values an order of magnitude greater than Expert 1 (Expert 2: 0.02, Expert 3: 0.03), and Trainees 1 and 2 achieving more comparable results (Trainee 1: 0.003, Trainee 2: 0.005). KM plots per observer are given in supplementary Fig. S3, but in summary, all observers produced a high-risk group with a 45.2–53.3% final risk of progression post-SRS, but the remaining three risk groups highly varied in amount of separation and final levels of progression risk.

The expert consensus appearance labelling showed a similar risk stratification to Expert 1 (Fig. 2b) at a comparable level of statistical significance (*p* = 0.009 vs. *p* = 0.007 for Expert 1). Risk groups 2 and 3 displayed slightly decreased separation for the expert consensus labelling, and the highest risk group (group 4) did not reach the same end level of risk at 18 months for the expert consensus (49.8%) compared to Expert 1 (54.4%).

### Qualitative appearance machine learning models

The radiomic Expert 1 appearance experiments showed varied accuracies when using radiomic features to perform each appearance labelling. The “homogeneous” results showed the highest AUC_0.632+_ = 0.92, while AUC_0.632+_ values of 0.77, 0.83, 0.88, and 0.72 were achieved for the “heterogeneous”, “cystic (simple)”, “cystic (complex)”, and “necrotic” labels respectively (full error metrics in Supplementary Fig. S4). As can be seen in Fig. 2c, when these qualitative appearance ML model results were used with the RPA model (as per Fig. 1b), the KM risk curves were negatively impacted considerably (*p* = 0.08).

**Figure 3:**
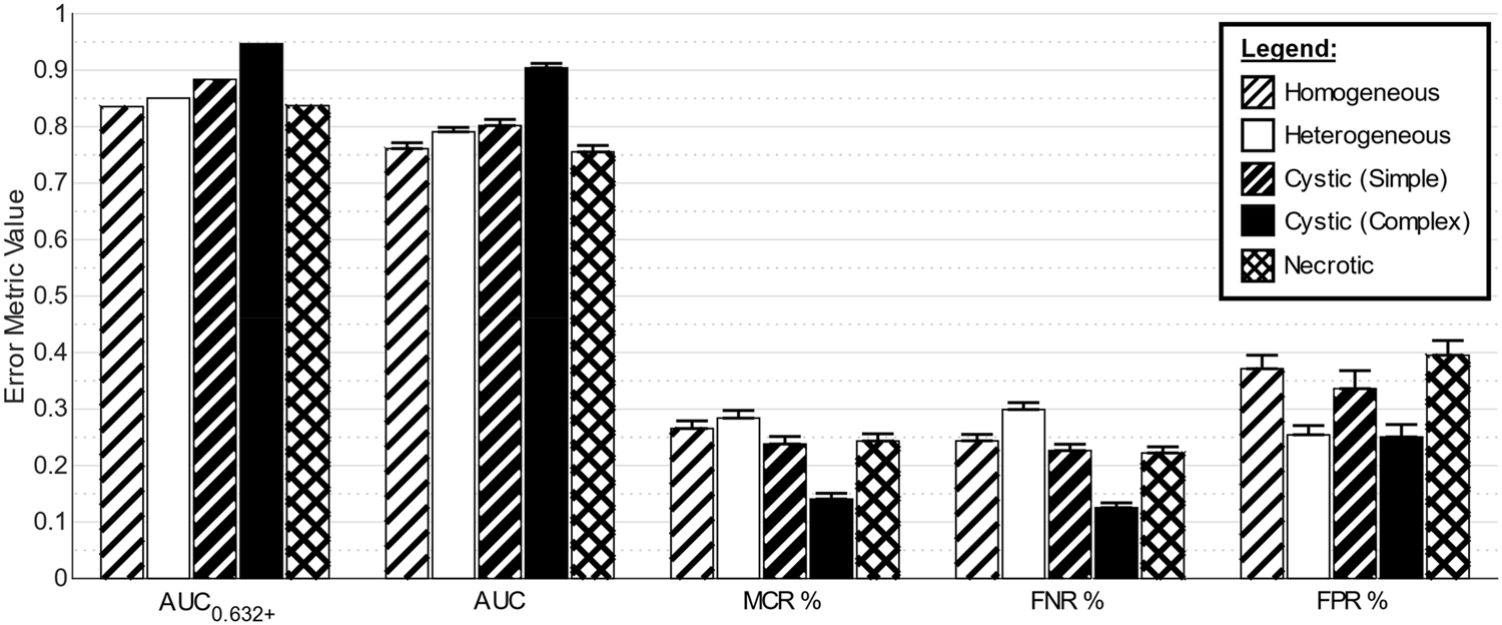
Error metrics from the radiomic consensus appearance experiments. As each appearance label (e.g. “homogeneous”) had specific models trained to make a binary labelling decision (e.g. “homogeneous” or “not homogeneous”), error metrics for each appearance label are presented. The error bars for the non-AUC_0.632+_ error metrics represent the 95% confidence interval of each value determined from the 250 bootstrapped resampling iterations.

The radiomic consensus appearance label experiments showed all appearances achieved AUC_0.632+_ ≥ 0.84 (Fig. 3). “Cystic (complex)” was labelled with the lowest MCR = 14.1% and highest AUC_0.632+_ = 0.95. “Homogeneous”, “heterogeneous”, and “necrotic” were labelled with similar accuracy (AUC_0.632+_ 0.84–0.85), with the highest MCR reported for “heterogeneous” (26.6%). The observed inaccuracy also negatively impacted the RPA model risk stratification (Fig. 2d), but statistical significance was retained (*p* = 0.04).

### Post-SRS progression machine learning models

The radiomic progression experiment directly predicted progression using only radiomic features with AUC_0.632+_ = 0.74. When using direct progression prediction instead of the qualitative appearance labels with the RPA model (as per Fig. 1c), the KM analysis revealed enhanced risk stratification (Fig. 2e, p = 0.0003). In particular, the analysis produced the risk groups with the lowest and highest risk of post-SRS progression (6.6% and 58.5%, respectively). Interestingly, risk group 2 was at higher risk for progression compared to group 3, the inverse of the RPA model.

The radiomic and clinical progression experiment achieved AUC_0.632+_ = 0.77, and when used to stratify BMs into risk groups (as per Fig. 1d), improved stratification was achieved compared to the RPA model results (Fig. 2f, p = 0.0006), with the results comparable to the previous stratification using the radiomic progression experiment (Fig. 2e).

### Post-SRS progression machine learning model interpretation

**Figure 4:**
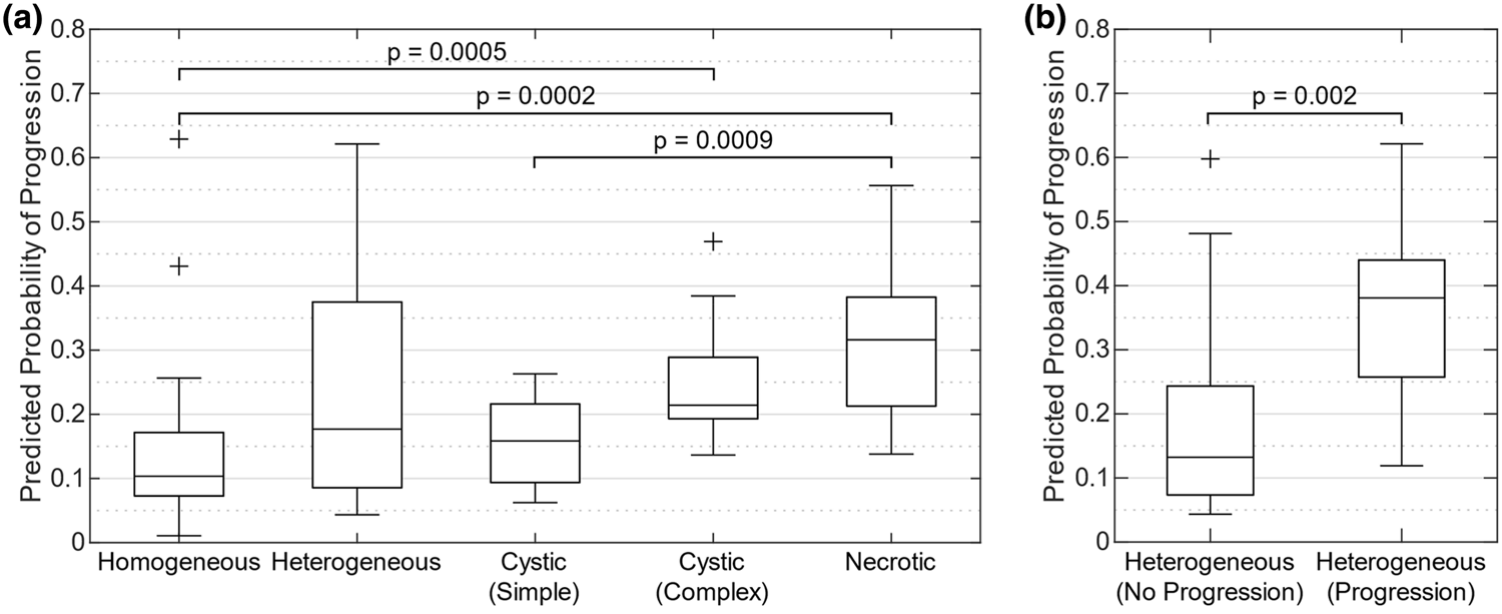
Comparison of the average predicted probability of progression for each BM from the radiomic progression experiment when grouped by qualitative appearance, as labelled by expert consensus. (**a**) Shows the comparison for each of the five appearance labels, with whiskers indicating the extreme values and outliers classified as being 1.5 × the interquartile range from the 25th or 75th percentile. The Kruskal–Wallis test across all groups found *p* = 0.0005, with ad hoc Wilcoxon rank-sum *p*-values shown on the plot for statistically significant comparisons (after Bonferroni correction). (**b**) Shows the splitting of the “Heterogeneous” distribution in (**a**) based on whether the BMs progressed or did not progress post-SRS, with the Wilcoxon rank-sum test *p*-value shown. Ad hoc Wilcoxon rank-sum tests between the “Heterogeneous (No Progression)” and “Heterogeneous (Progression)” distributions with the other appearance labels found that “Heterogeneous (No Progression)” was significantly different from the “Necrotic” BMs (*p* = 0.002), while “Heterogeneous (Progression)” was significantly different from the “Homogeneous” (*p* = 0.0009) and “Cystic (Simple)” (*p* = 0.001) BMs (significance determined after Bonferroni correction). The size and scale of the y-axes for (**a**,**b**) are equivalent to allow for simpler comparison.

We found that the average probability of post-SRS progression values per BM from the radiomic progression experiment were significantly different when compared across all appearance labels (Fig. 4a, Kruskal–Wallis *p* = 0.0005). BMs labelled “homogeneous” by expert consensus were associated with the lowest median progression probabilities, while “necrotic” had the highest. “Heterogeneous” demonstrated the largest interquartile range, nearly twice that of any other label. Ad hoc Wilcoxon rank-sum tests were then performed between pairs of labels, with three showing a significant difference (α = 0.005 after Bonferroni correction for 10 comparisons): “homogeneous” versus “cystic (complex)” or “necrotic”, and “cystic (simple)” versus “necrotic” (Fig. 4a).

The large “heterogeneous” probability interquartile range motivated further analysis between BMs that did progress post-SRS (+) and those that did not (−) for each appearance label. Wilcoxon rank-sum tests between the average predicted progression probabilities of + and − BMs for each appearance label found only “heterogeneous” labelled BMs to be significantly different, both with and without a Bonferroni correction for five comparisons. The “heterogeneous” BMs that did not progress had lower predicted probabilities of progression, while the BMs that did progress had a median predicted probability value greater than that of the “necrotic” BMs (Fig. 4b). Ad hoc Wilcoxon rank-sum tests between the + and − “heterogeneous” BMs and other appearance labels found that after a correction for eight comparisons, the − “heterogeneous” BMs were significantly different from the “necrotic” BMs, while + “heterogeneous” was significantly different from “homogeneous” and “cystic (simple)” (Fig. 4b).

Our feature importance analysis revealed 13 radiomic features that were highly important in the radiomic progression experiment. Ten of these features were second-order texture features, while two were first order statistical features, and one was a shape and size-based feature, as shown in Table 3. When compared to each radiomic consensus appearance experiment’s feature importance analysis, “necrotic” was found to have the highest median feature importance score across the same 13 features (0.70), with nine of the features also being in the top 13 most important features for labelling “necrotic” (see Table 3). “Homogeneous” and “cystic (complex)” both had median importance scores near 0.60, and the remaining appearance labels were lower again near 0.50.

**Table 3:** Feature importance ranks, scores, and ALE correlation comparison between the radiomic progression experiment and radiomic consensus appearance experiments.

Radiomic progression experiment				Radiomic consensus appearance experiments														
				Homogeneous			Heterogeneous			Cystic (Simple)			Cystic (Complex)			Necrotic		
Feature type	Feature name	Rank	Score	Rank	Score	ALE *ρ*	Rank	Score	ALE *ρ*	Rank	Score	ALE *ρ*	Rank	Score	ALE *ρ*	Rank	Score	ALE *ρ*
GLRLM	Gray-level non-uniformity	1	1.00	46	0.33	+ 0.29	48	0.21	+ 0.77	**11**	0.68	− 0.55	39	0.39	+ 0.92	33	0.44	+ 0.74
NGTDM	Contrast	2	0.95	24	0.51	− 0.09*	16	0.46	+ 0.31	30	0.50	+ 0.28	30	0.52	− 0.24	26	0.51	+ 0.78
GLCM	Inverse difference normalized	3	0.93	40	0.39	− 0.49	14	0.50	+ 0.82	14	0.61	− 0.88	50	0.31	+ 0.82	**6**	0.72	+ 0.96
GLSZM	Zone entropy	4	0.93	25	0.50	− 0.42	24	0.40	+ 0.25	19	0.57	+ 0.43	17	0.61	+ 0.99	**4**	0.74	+ 0.93
GLCM	Inverse variance	5	0.92	**7**	0.67	+ 0.98	**12**	0.52	− 0.42	24	0.53	+ 0.95	15	0.63	+ 0.79	25	0.51	+ 0.95
GLSZM	Zone percentage	6	0.88	44	0.36	− 0.74	57	0.18	+ 0.41	77	0.07	− 0.21	66	0.14	− 0.76	32	0.46	+ 0.80
GLDM	Dependence entropy	7	0.86	**10**	0.63	− 0.64	21	0.41	− 0.87	32	0.49	+ 0.83	**3**	**0.80**	+ 0.85	**7**	0.70	+ 0.98
Shape & Size	Surface volume ratio	8	0.83	**4**	**0.86**	− 0.39	31	0.36	+ 0.32	**5**	**0.85**	− 0.64	18	0.60	+ 0.72	**12**	0.59	− 0.29
GLRLM	Gray-level non-uniformity normalized	9	0.82	**8**	0.65	− 0.14*	**8**	0.56	+ 0.87	23	0.53	+ 0.72	**13**	0.64	− 0.43	**5**	0.73	+ 0.73
First Order	Kurtosis	10	0.81	**13**	0.60	− 0.67	**13**	0.51	+ 0.89	34	0.49	+ 0.81	**12**	0.64	+ 0.93	**1**	**1.00**	+ 0.83
GLCM	Correlation	11	0.81	56	0.23	− 0.53	63	0.14	− 0.99	42	0.38	+ 0.84	64	0.14	+ 0.90	**8**	0.70	+ 0.94
First Order	10th Percentile	12	0.81	17	0.59	+ 0.49	**1**	**1.00**	+ 0.34	**10**	**0.77**	− 0.27	**5**	0.74	− 0.04*	**2**	**0.87**	− 0.31
GLCM	Informational measure of correlation 2	13	0.78	14	0.59	+ 0.83	**2**	**0.79**	− 0.89	26	0.52	− 0.31	**11**	0.67	+ 0.73	**3**	**0.81**	+ 0.92
*Median values:*		*7*	*0.86*	*17*	*0.59*	− *0.39*	*16*	*0.46*	+ *0.32*	*24*	*0.53*	+ *0.28*	*17*	*0.61*	+ *0.79*	*7*	*0.70*	+ *0.83*
*Interquartile range values:*		*6.5*	*0.12*	*31.5*	*0.25*	*0.90*	*24.3*	*0.21*	*1.31*	*19.3*	*0.14*	*1.18*	*30.0*	*0.28*	*1.00*	*21.5*	*0.25*	*0.20*

“ALE *ρ*” refers to the Pearson correlation coefficient value between a given feature’s ALE plots for the radiomic progression experiment and a radiomic consensus appearance experiment. All ALE *ρ* values were significant at *p* < 0.05, except for the three values indicated (*). Bolded “Rank” and “Score” values are for emphasis only to show values within the same range as the radiomic progression experiment’s values (≥ 13 and ≥ 0.75 for importance rank and score, respectively).

Gray-Level Co-occurrence Matrix (GLCM), Gray-Level Run Length Matrix (GLRLM), Gray-Level Dependence Matrix (GLDM), Gray-Level Size Zone Matrix (GLSZM), Neighbouring Gray Tone Difference Matrix (NGTDM).

**Figure 5:**
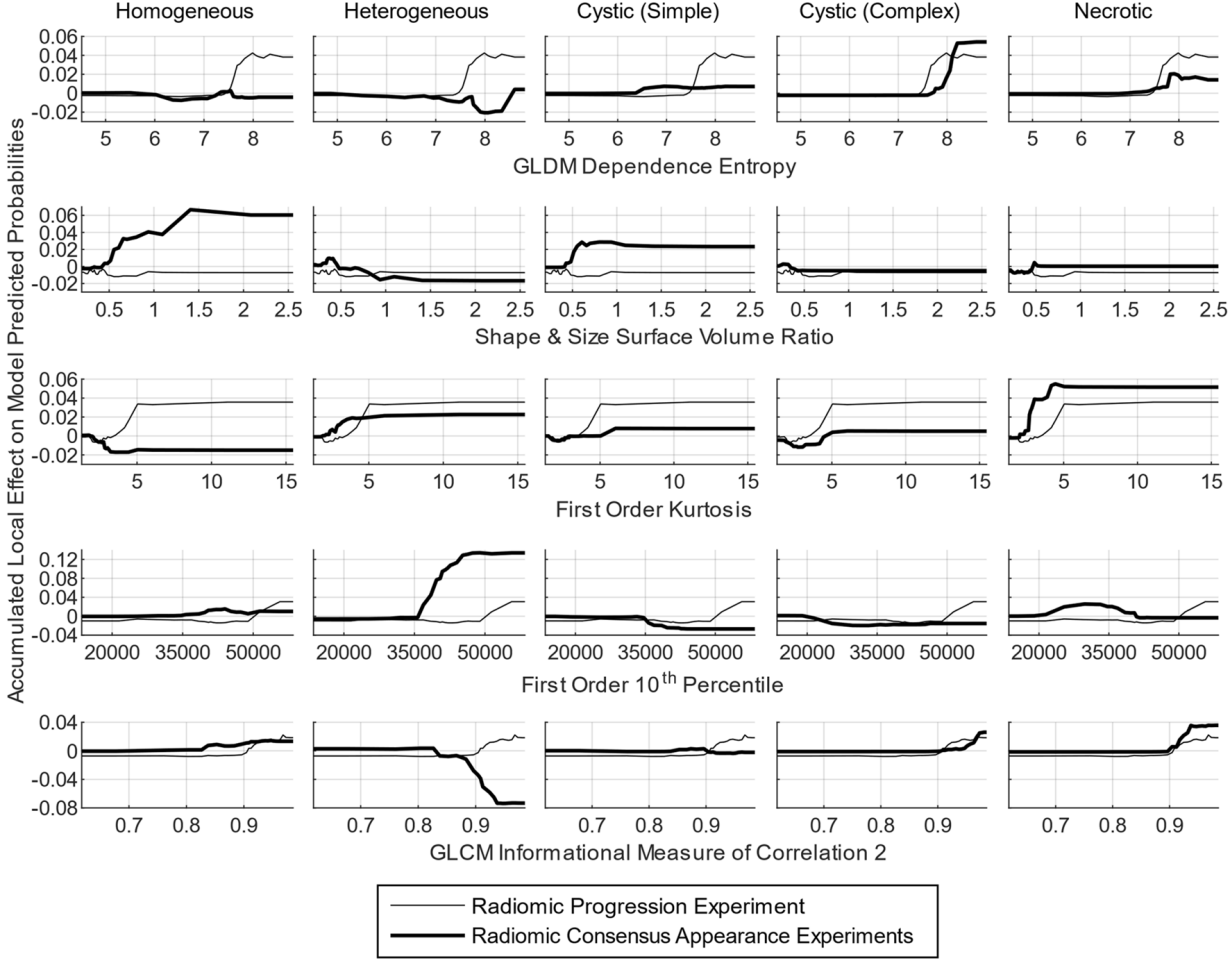
ALE plots comparing the effect of individual features on model predicted probabilities between the radiomic progression experiment and radiomic consensus appearance experiments. A single plot shows how as a feature’s value changes (x-axis) what the corresponding change in a model’s predicted positive label confidence is (y-axis), where a positive y-axis value indicates a shift in the model’s output towards predicting the positive label. Each row of ALE plots is for a single feature (as shown in the x-axes labels), with each plot comparing the feature’s effect on predicted probability of progression (same thin line across each plot in the same row), to the feature’s effect on labelling BMs with one of the appearance labels (thick lines). Each column of ALE plots therefore shows the effect for multiple features for one appearance label compared to predicting progression. The five features chosen to be displayed are all those that were highly important (importance score ≥ 0.75) for both the radiomic progression experiment and at least one radiomic appearance label experiment (see Table 3). ALE plots for the remaining highly important features not shown here can be found in supplementary Fig. S5. Each ALE plot consists of ALE values for 25 intervals chosen using 25 quantiles of a feature’s values across the entire dataset. For each ALE plot comparison, the Pearson correlation coefficient between the two curves is taken to produce the “ALE *ρ*” values in Table 3.

ALE plot analysis showed that the behaviour of many features used for labelling “necrotic” and “cystic (complex)” qualitatively matched that of features predicting progression, as shown in Fig. 5 and Supplementary Fig. S5. This observation was borne out quantitatively, with the “necrotic” and “cystic (complex)” labels having the highest median ALE *ρ* values of 0.83 and 0.79, respectively (Table 3). Only for the “homogeneous” label were the features found to be negatively correlated with predicting progression with median ALE *ρ* =  − 0.39. “Necrotic” also had the lowest interquartile range of ALE *ρ* values at 0.20, while “heterogeneous” had the largest at 1.31 (Table 3).

## Discussion

Our study found that interobserver variability of BM qualitative appearance labelling is high, but that an expert consensus produced similar risk stratification results to the original results using Expert 1’s labels and was more readily replicated by radiomics-based ML models (AUC_0.632+_ 0.84–0.94 across appearance labels). We also found that radiomics-based ML models directly predicting progression provided enhanced risk stratification compared to using qualitative appearance labelling, and that their radiomics signature was correlated with those of qualitative appearance labels.

The high interobserver variability in appearance labelling highlights the difficultly of using subjective, observer-based models. Across the observers, expertise level did not clearly impact variability, demonstrating the inherent uncertainty in the task. Differentiating between “heterogeneous” and “necrotic” appearances was the most difficult across all observers, likely due to both appearances presenting with areas of hypointensity in the T1w-CE MRI, and therefore requiring a subtle call of whether necrosis is present. Gutsche et al.’s study using three appearance labels (“homogeneous”, “heterogeneous”, or “ring-enhancing”) employed three observers with much higher interobserver agreement (κ = 0.75 vs. κ = 0.33–0.38)^16^. This is not entirely unexpected, as the three appearance labels used by Gutsche et al. did not require observers to make the difficult distinction between “heterogeneous” and “necrotic”. Differentiating between “heterogeneous” and “homogeneous” appearances was also very difficult, likely due to each observer choosing their own criteria on how much variability in the BM enhancement would define the border between “heterogeneous” and “homogeneous”.

The RPA model was somewhat resistant to interobserver variability, likely due to the first-level split based on the observer-independent SRS dose and fractionation and grouping of two or three appearance labels for each risk group. Despite this, the interobserver variability was high enough that model performance in turn varied widely, demonstrating the difficultly of using a single-observer model in practice. The highest-risk RPA group was the most stable, benefiting from “heterogeneous” and “necrotic” BMs being in the same risk group, which negated the effect of the high rate of interobserver disagreement observed specifically for this distinction. The lowest risk group was also quite stable, since this group contained “homogeneous” and “heterogeneous” BMs, again negating the effect of the high rate of interobserver disagreement between these two appearances. Despite the stability of these two risk groups, the overall predictive ability of the RPA model across all risk groups was unstable between observers. The expert consensus provided a technique to combat the interobserver variability, producing strong risk stratification, but applying such a technique to clinical practice could be impractical or labour intensive. Using multiple observers would also require further validation of the number, experience, and clinical speciality of the observers necessary for clinical implementation. Currently no standardized protocol for scoring the qualitative appearance of BMs exist, and so developing such a protocol or incorporating hypoxia-specific imaging techniques^37^ could possibly reduce interobserver variability, but these methods would need to be developed and validated.

Our radiomic Expert 1 and consensus appearance experiments’ results showed that replicating observer-based appearance labelling with radiomics-based ML was not feasible. This resulted in the poorest BM risk stratification due to ML model inaccuracy, which also highlighted the RPA’s model sensitivity to this inaccuracy. Our interobserver variability results showed that BM appearance across labels can be highly similar, and so ML models also appear to struggle to recognize patterns that are specifically indicative of each appearance label. The results from replicating Expert 1 again showed the difficulty in distinguishing “heterogeneous” and “necrotic” BMs, with these labels reporting the lowest ML model accuracy. Using the expert consensus appearance labels did result in more accurate replication by ML models, likely due to more consistent appearance labelling compared to a single observer, but the remaining inaccuracy still severely compromised the risk stratification. Having an objective radiomics model that produces highly interpretable qualitative appearance labels would be ideal for clinical use, but our results demonstrate that such a system is not currently feasible using the techniques we employed.

Using radiomics-based ML models to directly predict BM progression presents an alternative approach to outcome prediction that our results show is superior for post-SRS progression risk stratification compared to observer-based techniques. While Gutsche et al.^16^ and Kawahara et al.^18^ showed similar superiority of ML models to the three-way qualitative appearance labelling scheme, our study provides the first comparison of ML models to a more comprehensive five-way appearance labelling scheme and comparison to a more clinically applicable qualitative appearance predictive model built on multi-variate data. These results are also the first to compare radiomics-based ML and qualitative appearance models using risk stratification KM analysis, instead of the AUC and MCR error metrics typically reported in ML studies. Risk stratification analysis not only allows direct comparison to non-ML studies, but is also highly clinically interpretable.

As we used ML models using only radiomic features to both produce qualitative appearance labels and the probability of post-SRS progression, by comparing the two approaches in the same RPA model (Fig. 1b vs. c), the effect of constraining the ML models to use the five qualitative appearance labels could be seen. Our results clearly show that allowing the ML model to find the optimal radiomic signature to predict progression independently of the five qualitative appearance labels led to the greatest stratification of risk. This effect was so pronounced that the ML model’s risk group 2 was at higher risk for progression post-SRS compared to risk group 3, whereas the original RPA model indicated this would not occur when basing decisions only on the qualitative appearance labels.

The results from the radiomic and clinical progression experiment present the most desirable risk stratification for clinical use (Figs. 1d, 2f). The low-risk groups (1 and 2) indicate a population of BMs that would not benefit from SRS treatment modification. Risk groups 3 and 4 represent BMs that may benefit from SRS dose escalation (if feasible with respect to possible adjacent organs-at-risk), with group 3 specific to patients with a favourable prognosis, as group 3’s risk of progression was less that half that of group 4 at 12 months. This study’s results therefore motivate the use of ML techniques moving forward in research and clinical translation, as ML techniques have been shown to provide benefits in accuracy and consistency for outcome prediction compared to traditional qualitative appearance techniques.

The enhanced risk stratification of ML models directly predicting post-SRS progression is encouraging, but they do require interpretation to provide insight into their operation. Our results comparing the radiomic progression experiment’s predicted probability of progression across qualitative appearance labels offers some of these insights (Fig. 4). It appears from this analysis that our ML models trained across bootstrapped iterations, while free to find any radiomic signature predictive of progression, discovered an optimal radiomic signature related to the qualitative appearance labels. The ML models independently discovered that “necrotic” BMs were at the highest risk of post-SRS progression and “homogeneous” BMs were at the lowest risk, which agrees with previous studies’ hypotheses that implicated hypoxic conditions within the BM in causing reduced SRS effectiveness^11,12^. The ML models also found radiomic differences in the “heterogeneous” BMs that successfully separated the BMs that progressed post-SRS and those that did not. This indicates that there are additional radiomic differences within the “heterogeneous” BMs that are useful for predicting response, but are not readily visually distinguished. Given the similarity of the predicted probability of progression distributions between the “necrotic” BMs and the “heterogeneous” BMs found to progress, we speculate that there could be a BM population labelled as “heterogeneous” that are hypoxic enough to negatively impact SRS outcomes, but do not yet qualitatively appear necrotic in T1w-CE MRI.

To provide further interpretation of the ML models, we also analyzed if the radiomic progression experiment's radiomic signature was correlated with those of the radiomic consensus appearance experiments. Our results (Table 3, Fig. 5) indicated that the progression radiomics signature was most closely correlated with the “necrotic” radiomics signature, both in terms of feature importance scores and correlation between ALE plots. This shows that the radiomics progression experiment not only independently found necrotic BMs to be at highest risk for progression, but also used a similar radiomic signature to do so. The other qualitative appearance radiomic signatures have a lower similarity with the progression radiomic signature in terms of feature importance, but their ALE correlation values generally match the results from Fig. 4 (e.g. “homogeneous” BMs had the lowest predicted probability of progression and the “homogeneous” radiomic signature was also the most anti-correlated with the progression radiomic signature). Furthermore, the “heterogeneous” radiomic signature had the largest interquartile range of ALE correlation values, showing that this radiomic signature was both highly correlated and anti-correlated with the progression radiomic signature. This matches our previous finding of two distinct subpopulations of “heterogeneous” BMs, which could then lead to a “heterogeneous” radiomic signature with mixed correlation with the progression radiomic signature.

These model interpretation results promisingly indicate a possible connection between the post-SRS progression radiomic signature and necrosis, but the ultimate strength of this connection is limited by a few factors. First, the labelling of BMs as “necrotic” (or any other appearance label), while performed by expert consensus, is unlikely to be exactly reflective of the true underlying biology. Second, the radiomic consensus appearance experiments did not produce perfectly accurate models, and third, the radiomic progression experiment also had model inaccuracy. Lastly, the ALE correlation values only demonstrate correlations between progression and qualitative appearance for individual radiomic features, and so interpreting the correlation values across all features can be difficult. Therefore, while our results are informative, they are not conclusive.

Previous ML studies interpreting their models’ operation have mostly found texture-based radiomic features to be predictive of SRS response (as opposed to shape/size or first-order statistical features), but further interpretation of these features is difficult^15–25,28^. Some studies have considered radiomic features from different MRI sequences and ROIs (tumour core, peri-tumoural regions, or surrounding edema), and so some interpretation of these results is possible^16,17,19–21,25,28^. Studies using convolutional neural networks can similarly analyze which data the network was focusing its “attention” when making predictions^26,27^. Across these studies, however, there is disagreement on the relative value of both different MRI sequences and ROIs. Gutsche et al. found all 10 of their important radiomic features had significantly different values when grouped between “homogeneous”, “heterogeneous”, and “ring-enhancing” BMs^16^. While this analysis technique provides insight into the individual radiomic features used by a ML model, ML models incorporate complex and non-linear relationships between these features. Therefore, the analysis we provide of interpreting the model as a whole through analysis of the model predicted probabilities and ALE plots is critical to provide a more comprehensive model interpretation. This model interpretation and connection to underlying biological processes not only provides a more intuitive understanding of ML model operation, but could also be used to assess new ML techniques or test biological theories developed for SRS outcome prediction.

Our study’s results need to be considered in the context of our methodology’s limitations. First, our study was retrospective, and so our study sample inherently reflects only the population of BM patients treated at the AUMC during the time of data collection. As our patient sample was treated between 2003 and 2011, our results need to be confirmed on patients treated more recently due to advancements in imaging technology, SRS techniques, and systemic therapies. Second, due to our small dataset size, we used a bootstrapped resampling ML experimental design and could not perform external validation. While our methods took care to prevent leakage of the testing dataset into the model training process, an external validation dataset is required to confirm our results. Lastly, our studied endpoint was both non-standard and radiographically defined. As our dataset was acquired before the introduction of the standardized RANO-BM protocol^31^, it relies on non-standard BM measurements. While our measurements were more comprehensive than those mandated by RANO-BM^31^, they are unfortunately not directly comparable to other studies. Furthermore, our radiographically-defined endpoint is susceptible to pseudo-progression, which cannot be fully controlled for in a retrospective study. Therefore, prospective studies with well-defined protocols for controlling for pseudo-progression are required.

While confirmation of our T1w-CE MRI interpretability results is critical, it is also important to extend our analysis techniques to studies using other MRI sequences and ROIs, especially given their reported predictive value. Such studies would require the development and validation of qualitative appearance labels specific to each MRI sequence and ROI to ensure interpretability is grounded in clinically and biologically relevant explanations. Our ML model interpretation results motivate defining the ground truth biological status of a BM using pathological data. Pathological data would provide a more reliable source of comparison for radiomic signatures, versus the qualitative MRI analysis used in in this study. A joint BM MRI radiomic and pathology study is ultimately required to provide definitive explanations of MRI radiomics-based ML models. Such a study is highly feasible given that T1w-CE MRI is routinely collected before most BM surgical resections, and a retrospective study may even be possible if banked or digitized tissue samples are available.

In conclusion, we have compared observer-based qualitative appearance models to machine learning models using quantitative MRI radiomic features for predicting brain metastasis response to stereotactic radiosurgery. We showed that the interobserver variability of appearance labelling is high and negatively impacts predictive model performance. Trying to replicate appearance labelling with machine learning models was shown to be possible, but did not lead to high outcome prediction accuracy, which therefore led to investigating using machine learning models that directly predict outcomes. These models that directly predicted outcomes were found to outperform observer-based models, motivating the use of machine learning models in future clinical translation. We also provided interpretation of our radiomics models, showing that their operation was related to qualitative appearance labels, with “necrotic” metastases and a subset of “heterogeneous” metastases predicted to have the highest probability of progression post-treatment. Our results therefore provide a necessary step in model interpretation that is required for the eventual clinical translation of these models that will allow for optimized treatment outcomes for brain metastasis patients.

### Supplementary Information

Supplementary Information.

## Data Availability

The progression labels, qualitative appearance labels, and model prediction probabilities from each reported experiment needed to replicate this study’s analysis are available for use at the following URL: https://github.com/baines-imaging-research-laboratory/radiomics-for-srs-model-interpretability-data-share.

## Code availability

All the computer code used for this study’s machine learning experiments, analysis and figure generation are available online at: https://github.com/baines-imaging-research-laboratory/radiomics-for-srs-model-interpretability-code.

## References

[1] Nayak L, Lee EQ, Wen PY (2012). Epidemiology of brain metastases. Curr. Oncol. Rep..

[2] Sperduto PW (2020). Survival in patients with brain metastases: Summary report on the updated diagnosis-specific graded prognostic assessment and definition of the eligibility quotient. J. Clin. Oncol..

[3] Le Rhun E (2021). EANO–ESMO Clinical Practice Guidelines for diagnosis, treatment and follow-up of patients with brain metastasis from solid tumours. Ann. Oncol..

[4] Vogelbaum MA (2022). Treatment for brain metastases: ASCO-SNO-ASTRO guideline. Neuro. Oncol..

[5] Chao ST (2018). Stereotactic radiosurgery in the management of limited (1–4) brain metasteses: Systematic review and international stereotactic radiosurgery society practice guideline. Neurosurgery.

[6] Sneed PK (2015). Adverse radiation effect after stereotactic radiosurgery for brain metastases: Incidence, time course, and risk factors. J. Neurosurg..

[7] Blonigen BJ (2010). Irradiated volume as a predictor of brain radionecrosis after linear accelerator stereotactic radiosurgery. Int. J. Radiat. Oncol..

[8] Nieder C, Berberich W, Schnabel K (1997). Tumor-related prognostic factors for remission of brain metastases after radiotherapy. Int. J. Radiat. Oncol. Biol. Phys..

[9] Kim YS, Kondziolka D, Flickinger JC, Lunsford LD (1997). Stereotactic radiosurgery for patients with nonsmall cell lung carcinoma metastatic to the brain. Cancer.

[10] Peterson AM, Meltzer CC, Evanson EJ, Flickinger JC, Kondziolka D (1999). MR imaging response of brain metastases after gamma knife stereotactic radiosurgery. Radiology.

[11] Goodman KA (2001). Relationship between pattern of enhancement and local control of brain metastases after radiosurgery. Int. J. Radiat. Oncol. Biol. Phys..

[12] Shiau C-Y (1997). Radiosurgery for brain metastases: Relationship of dose and pattern of enhancement to local control. Int. J. Radiat. Oncol. Biol. Phys..

[13] Naoi Y, Maehara T, Cho N, Katayama H (1999). Stereotactic radiosurgery for brain metastases using a linac system: Evaluation of initial local response by imaging. Radiat. Med..

[14] Rodrigues G, Zindler J, Warner A, Lagerwaard F (2013). Recursive partitioning analysis for the prediction of stereotactic radiosurgery brain metastases lesion control. Oncologist.

[15] DeVries DA (2022). Performance sensitivity analysis of brain metastasis stereotactic radiosurgery outcome prediction using MRI radiomics. Sci. Rep..

[16] Gutsche R (2022). Radiomics outperforms semantic features for prediction of response to stereotactic radiosurgery in brain metastases. Radiother. Oncol..

[17] Jiang ZK (2022). Multimodality MRI-based radiomics approach to predict the posttreatment response of lung cancer brain metastases to gamma knife radiosurgery. Eur. Radiol..

[18] Kawahara D, Tang X, Lee CK, Nagata Y, Watanabe Y (2021). Predicting the local response of metastatic brain tumor to gamma knife radiosurgery by radiomics with a machine learning method. Front. Oncol..

[19] Liao CY (2021). Enhancement of radiosurgical treatment outcome prediction using MRI radiomics in patients with non-small cell lung cancer brain metastases. Cancers.

[20] Mouraviev A (2020). Use of radiomics for the prediction of local control of brain metastases after stereotactic radiosurgery. Neuro Oncol..

[21] Wang HS (2021). Predicting local failure of brain metastases after stereotactic radiosurgery with radiomics on planning MR images and dose maps. Med. Phys..

[22] Du P (2023). Prediction of treatment response in patients with brain metastasis receiving stereotactic radiosurgery based on pre-treatment multimodal MRI radiomics and clinical risk factors: A machine learning model. Front. Oncol..

[23] Carloni G (2023). Brain metastases from NSCLC treated with stereotactic radiotherapy: Prediction mismatch between two different radiomic platforms. Radiother. Oncol..

[24] Mulford K (2021). A radiomics-based model for predicting local control of resected brain metastases receiving adjuvant SRS. Clin. Transl. Radiat. Oncol..

[25] Jaberipour M, Soliman H, Sahgal A, Sadeghi-Naini A (2021). A priori prediction of local failure in brain metastasis after hypo-fractionated stereotactic radiotherapy using quantitative MRI and machine learning. Sci. Rep..

[26] Jalalifar SA, Soliman H, Sahgal A, Sadeghi-Naini A (2022). Predicting the outcome of radiotherapy in brain metastasis by integrating the clinical and MRI-based deep learning features. Med. Phys..

[27] Jalalifar SA, Soliman H, Sahgal A, Sadeghi-Naini A (2022). A self-attention-guided 3D deep residual network with big transfer to predict local failure in brain metastasis after radiotherapy using multi-channel MRI. IEEE J. Transl. Eng. Heal. Med..

[28] Karami E (2019). Quantitative MRI biomarkers of stereotactic radiotherapy outcome in brain metastasis. Sci. Rep..

[29] van Griethuysen JJM (2017). Computational radiomics system to decode the radiographic phenotype. Cancer Res..

[30] Gillies RJ, Kinahan PE, Hricak H (2015). Radiomics: Images are more than pictures, they are data. Radiology.

[31] Lin NU (2015). Response assessment criteria for brain metastases: Proposal from the RANO group. Lancet Oncol..

[32] Collewet G, Strzelecki M, Mariette F (2004). Influence of MRI acquisition protocols and image intensity normalization methods on texture classification. Magn. Reson. Imaging.

[33] Efron B, Tibshirani R (1997). Improvements on cross-validation: The .632+ bootstrap method. J. Am. Stat. Assoc..

[34] Fedorov A (2012). 3D Slicer as an image computing platform for the Quantitative Imaging Network. Magn. Reson. Imaging.

[35] Molnar, C. *Interpretable Machine Learning*. https://christophm.github.io/interpretable-ml-book (2022).

[36] Apley DW, Zhu J (2020). Visualizing the effects of predictor variables in black box supervised learning models. J. R. Stat. Soc. Ser. B Stat. Methodol..

[37] Horsman MR, Mortensen LS, Petersen JB, Busk M, Overgaard J (2012). Imaging hypoxia to improve radiotherapy outcome. Nat. Rev. Clin. Oncol..

