# Supplementary material for: Predicting stereotactic radiosurgery outcomes with multi-observer qualitative appearance labelling versus MRI radiomics
Source: Sci Rep. 2023 Nov 28;13:20977. doi: 10.1038/s41598-023-47702-8 (PMC10684869; doi:10.1038/s41598-023-47702-8)
Supplement: Supplementary file 1 — Supplementary Information. [file 41598_2023_47702_MOESM1_ESM.pdf]

Predicting Stereotactic Radiosurgery Outcomes with Multi-Observer Qualitative Appearance Labelling versus MRI Radiomics

Supplementary Material

David A. DeVries[1,2,*], Terence Tang[3], Ali Albweady[4], Andrew Leung[5], Joanna Laba[3,6], Carol Johnson[2], Frank Lagerwaard[7], Jaap Zindler[8,9], George Hajdok[1], Aaron D. Ward[1,2,6]

[1]Western University, Department of Medical Biophysics, London, N6A 3K7, Canada
[2]Gerald C. Baines Centre, London Health Sciences Centre, London, N6A 5W9, Canada
[3]London Health Sciences Centre, Department of Radiation Oncology, London, N6A 5W9, Canada
[4]Unaizah College of Medicine and Medical Sciences, Department of Radiology, Qassim University, Buraidah, 56219, Saudi Arabia
[5]Western University, Department of Medical Imaging, London, N6A 3K7, Canada
[6]Western University, Department of Oncology, London, N6A 3K7, Canada
[7]Amsterdam University Medical Centre, Department of Radiation Oncology, Amsterdam, 1081, The Netherlands
[8]Haaglanden Medical Centre, Department of Radiation Oncology, Den Hague, 2512VA, The Netherlands
[9]Holland Proton Centre, Delft, 2629JA, The Netherlands
*ddevrie8@uwo.ca

Clinical Features	# Patients	# BMs (% Progression)
Sex		
Female	55	66 (21.2%)
Male	44	57 (22.8%)
Age		
Median (Range)	58.0 (38.4-86.0) years	
Primary Cancer Active		
Yes	44	55 (9.1%)
No	55	68 (32.4%)
Primary Cancer Site		
Lung	59	70 (12.9%)
Breast	10	14 (35.7%)
Renal	10	15 (13.3%)
Colorectal	8	10 (40.0%)
Skin	8	9 (66.7%)
Other	4	5 (20.0%)
Primary Cancer Histology		
Adenocarcinoma	49	65 (20.0%)
NSCLC	31	36 (11.1%)
Melanoma	8	9 (66.7%)
Squamous Carcinoma	7	8 (50.0%)
Other	4	5 (0.0%)
Extracranial Systemic Metastases		
Yes	39	50 (20.0%)
No	60	73 (23.3%)
Systemic Therapy Status		
Radical	51	10 (20.0%)
Palliative	41	60 (31.7%)
None	7	53 (11.3%)
Neurological Symptoms Corticosteroid Response		
Fully resolved	48	31 (16.1%)
Improvement	7	4 (50.0%)
Limited improvement	4	56 (25.0%)
No improvement	26	11 (9.1%)
Unknown	14	21 (23.8%)
ECOG Performance Score		
0	31	39 (17.9%)
1	60	73 (21.9%)
2	6	9 (33.3%)
3	2	2 (50.0%)
GTV Volume		
Median (Range)	3.07 (0.02-30.23) cc	
BM Location		
Supratentorial	-	96 (22.9%)
Infratentorial	-	27 (18.5%)
SRS Prescription		
15 Gy in 1 fraction	-	5 (0.0%)
18 Gy in 1 fraction	-	36 (30.6%)
21 Gy in 1 fraction	-	72 (13.9%)
24 Gy in 3 fractions	-	10 (60.0%)

Table S1. Clinical feature distributions for number of BMs, BMs progressing post-SRS, and patients (where applicable) for the study sample. The “Neurological Symptoms Corticosteroid Response” feature qualitatively scores the improvement of neurological symptoms after the administration of corticosteroids. Abbreviations: NSCLC (non-small cell lung cancer), Eastern Cooperative Oncology Group (ECOG), GTV (gross tumour volume)

Scanner Model and Field Strength	Acquisition Orientation	Voxel Size (mm^3)	# Patients	# BMs (% Progression)
Siemens Magnetom Vision *(1.5 T)*	Sagittal	1×1×1.5	35	39 (28.2%)
Siemens Avanto *(1.5 T)*	Sagittal	0.5×0.5×1	30	37 (13.5%)
	Axial	0.5×0.5×2	5	8 (0.0%)
Siemens Magnetom Expert *(1.0 T)*	Sagittal	1×1×1.5	21	29 (31.0%)
Siemens Sonata *(1.5 T)*	Sagittal	1×1×1.5	5	5 (40.0%)
	Axial	1×1×1.5	1	1 (0.0%)
	Axial	1×1×2	1	3 (0.0%)
General Electric Signa HDxt *(1.5 T)*	Sagittal	1×1×1.5	1	1 (0.0%)

Table S2. Number of patients and BMs scanned by each of the MR scanner models and acquisition parameter configurations. Siemens (Erlangen, Germany); General Electric (Chicago, USA)

#	Feature Type	Feature Name	#	Feature Type	Feature Name
1	First Order	10th Percentile	57	GLRLM	Gray-Level Non-Uniformity
2	First Order	90th Percentile	58	GLRLM	Gray-Level Non-Uniformity Normalized
3	First Order	Energy	59	GLRLM	Gray-Level Variance
4	First Order	Entropy	60	GLRLM	High Gray-Level Run Emphasis
5	First Order	Interquartile Range	61	GLRLM	Long Run Emphasis
6	First Order	Kurtosis	62	GLRLM	Long Run High Gray-Level Emphasis
7	First Order	Maximum	63	GLRLM	Long Run Low Gray-Level Emphasis
8	First Order	Mean Absolute Deviation	64	GLRLM	Low Gray-Level Run Emphasis
9	First Order	Mean	65	GLRLM	Run Entropy
10	First Order	Median	66	GLRLM	Run Length Non-Uniformity
11	First Order	Minimum	67	GLRLM	Run Length Non-Uniformity Normalized
12	First Order	Range	68	GLRLM	Run Percentage
13	First Order	Robust Mean Absolute Deviation	69	GLRLM	Run Variance
14	First Order	Root Mean Squared	70	GLRLM	Short Run Emphasis
15	First Order	Skewness	71	GLRLM	Short Run High Gray-Level Emphasis
16	First Order	Total Energy	72	GLRLM	Short Run Low Gray-Level Emphasis
17	First Order	Uniformity	73	GLDM	Dependence Entropy
18	First Order	Variance	74	GLDM	Dependence Non-Uniformity
19	Shape & Size	Elongation	75	GLDM	Dependence Non-Uniformity Normalized
20	Shape & Size	Flatness	76	GLDM	Dependence Variance
21	Shape & Size	Least Axis Length	77	GLDM	Gray-Level Non-Uniformity
22	Shape & Size	Major Axis Length	78	GLDM	Gray-Level Variance
23	Shape & Size	Maximum 2D Diameter Column	79	GLDM	High Gray-Level Emphasis
24	Shape & Size	Maximum 2D Diameter Row	80	GLDM	Large Dependence Emphasis
25	Shape & Size	Maximum 2D Diameter Slice	81	GLDM	Large Dependence High Gray-Level Emphasis
26	Shape & Size	Maximum 3D Diameter	82	GLDM	Large Dependence Low Gray-Level Emphasis
27	Shape & Size	Mesh Volume	83	GLDM	Low Gray-Level Emphasis
28	Shape & Size	Minor Axis Length	84	GLDM	Small Dependence Emphasis
29	Shape & Size	Sphericity	85	GLDM	Small Dependence High Gray-Level Emphasis
30	Shape & Size	Surface Area	86	GLDM	Small Dependence Low Gray-Level Emphasis
31	Shape & Size	Surface Volume Ratio	87	GLSZM	Gray-Level Non-Uniformity
32	Shape & Size	Voxel Volume	88	GLSZM	Gray-Level Non-Uniformity Normalized
33	GLCM	Autocorrelation	89	GLSZM	Gray-Level Variance
34	GLCM	Cluster Prominence	90	GLSZM	High Gray-Level Zone Emphasis
35	GLCM	Cluster Shade	91	GLSZM	Large Area Emphasis
36	GLCM	Cluster Tendency	92	GLSZM	Large Area High Gray-Level Emphasis
37	GLCM	Contrast	93	GLSZM	Large Area Low Gray-Level Emphasis
38	GLCM	Correlation	94	GLSZM	Low Gray-Level Zone Emphasis
39	GLCM	Difference Average	95	GLSZM	Size Zone Non-Uniformity
40	GLCM	Difference Entropy	96	GLSZM	Size Zone Non-Uniformity Normalized
41	GLCM	Difference Variance	97	GLSZM	Small Area Emphasis
42	GLCM	Inverse Difference	98	GLSZM	Small Area High Gray-Level Emphasis
43	GLCM	Inverse Difference Moment	99	GLSZM	Small Area Low Gray-Level Emphasis
44	GLCM	Inverse Difference Moment Normalized	100	GLSZM	Zone Entropy
45	GLCM	Inverse Difference Normalized	101	GLSZM	Zone Percentage
46	GLCM	Informational Measure of Correlation 1	102	GLSZM	Zone Variance
47	GLCM	Informational Measure of Correlation 2	103	NGTDM	Busyness
48	GLCM	Inverse Variance	104	NGTDM	Coarseness
49	GLCM	Joint Average	105	NGTDM	Complexity
50	GLCM	Joint Energy	106	NGTDM	Contrast
51	GLCM	Joint Entropy	107	NGTDM	Strength
52	GLCM	Maximal Correlation Coefficient			
53	GLCM	Maximum Probability			
54	GLCM	Sum Average			
55	GLCM	Sum Entropy			
56	GLCM	Sum Squares			

Table S3. Complete catalogue of the 107 radiomic features included within the study. All features were computed on the pre-treatment T1w-CE MRI, with complete documentation of the features provided by the PyRadiomics project (https://pyradiomics.readthedocs.io/en/latest/features.html). Abbreviations: Gray-Level Co-occurrence Matrix (GLCM), Gray-Level Run Length Matrix (GLRLM), Gray-Level Dependence Matrix (GLDM), Gray-Level Size Zone Matrix (GLSZM), Neighbouring Gray Tone Difference Matrix (NGTDM).

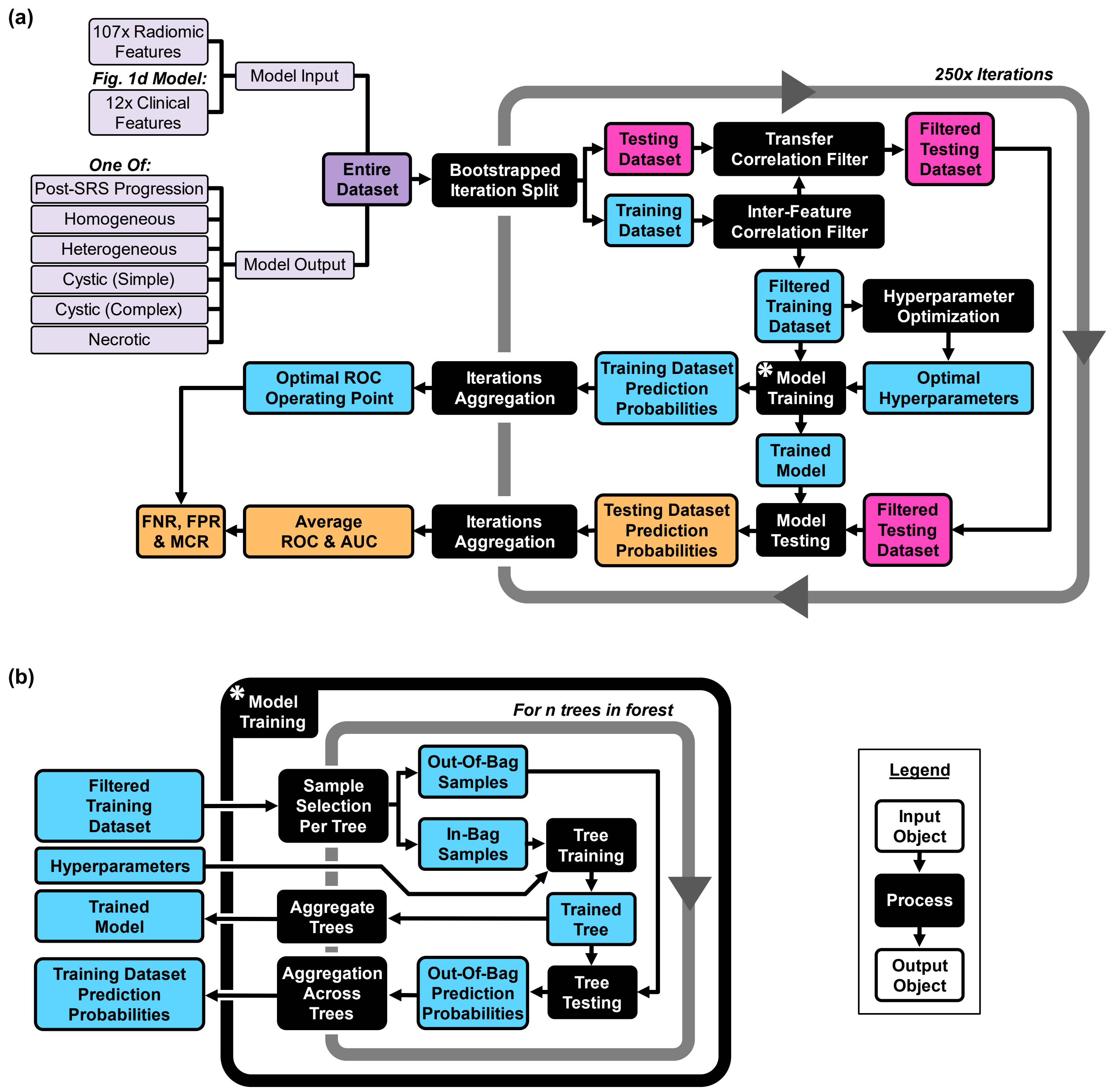

Figure S1. Schematic diagram of the machine learning experimental method technique used in the study. (a) shows the overall experimental method, while (b) provides enhanced detail of the model training to show how the out-of-bag samples from each tree in the random decision forest are used to produce the aggregated training dataset prediction probabilities. The colouring of the objects in the diagram is used to illustrate the separation of the entire dataset into training and testing datasets, the isolation of objects derived from each of the datasets during feature filtering and hyperparameter optimization (to prevent overfitting), and the recombination of objects from the datasets only during model testing and error metrics calculation. The inter-feature correlation filter used hierarchical clustering on the training dataset alone to determine groups of correlated features with a correlation coefficient > 0.8. For each group of correlated features, the feature most strongly correlated to the model output was retained. This filtering of features was then transferred to the testing dataset, ensuring the testing dataset did not inform the selected features. Depending on whether the intent of the machine learning experiment was to predict the probability of progression or to replicate an observer's qualitative appearance labelling, one of the six different model outputs would be provided, as shown. All experiments used the set of 107 radiomic features (Table S3), but only the radiomic and clinical progression experiment (Fig. 1d) included the set of 12 clinical features (Table S1). Table S4 provides further detail on the hyperparameter optimization, while Figure S2 shows how this machine learning experiment template was applied to produce the reported results and analysis.

Hyperparameter	Optimization Domain	Optimization Domain Search Transform
Number of trees	[10, 1000]	logarithmic
Number of features to sample	[1, number of features]	linear
Minimum leaf size	[1, number of features / 2]	logarithmic
Maximum number of decision splits	[1, number of samples – 1]	logarithmic
Feature selection	curvature, interaction curvature	categorical
Decision split criterion	Gini’s diversity index, deviance	categorical
	**Value**	**Justification**
In-bag fraction	1	produces in-bag dataset that is the same size as the training dataset
Sample with replacement	on	allows for out-of-bag samples to be reserved for evaluating trained model using the training dataset
Cost per SRS response	equal for each response	false negatives and positives given equal cost
Prior	empirical	allows priors to be optimized for the study population
Algorithm for categorical features	exact	all combinations of categories for categorical features investigated at decision splits
Merge leaves	off	leaf merging not needed as trees are not pruned
Prune	off	tree pruning not needed as the maximum number of decision splits hyperparameter was optimized
Surrogate decision splits	10	not all surrogate splits investigated to decrease model training time
Weights	equal for each sample	all samples given equal importance during training

Table S4. Hyperparameters for the random decision forest model used. For hyperparameters that underwent optimization, the optimization domain and search transform are provided. Numerical domains are indicated with minimum and maximum values in square brackets. Hyperparameter optimization was performed using 50 iterations of Bayesian optimization using the expected-improvement-plus acquisition function. The AUC on the out-of-bag samples was used as the optimization objective function. For hyperparameters that were not optimized, their value and justification are provided. For further descriptions of the hyperparameters, see the documentation for the *TreeBagger* function provided in Matlab 2019b.

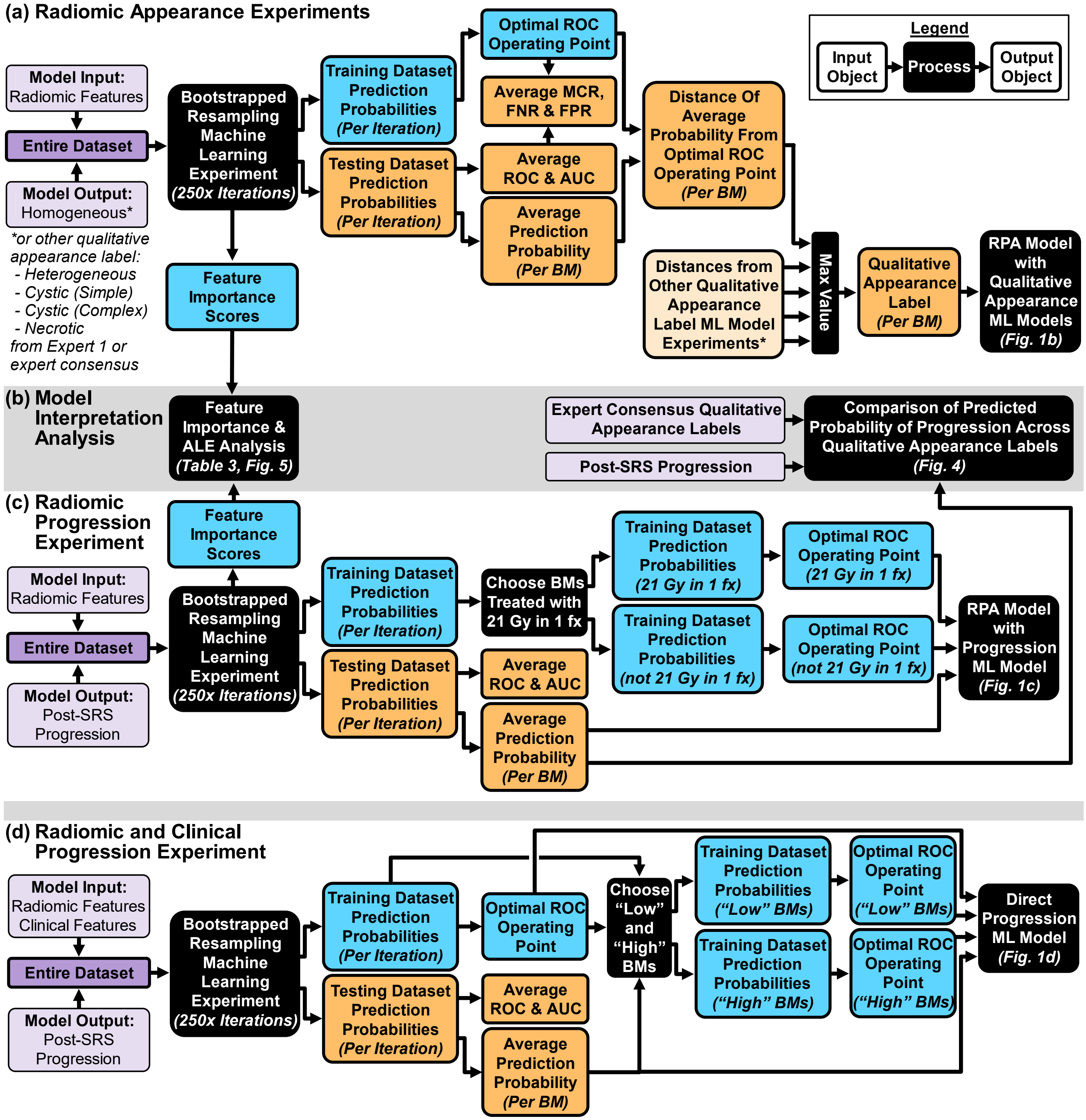

Figure S2. Schematic of the ML experiments and analysis associated with (a) the radiomic appearance experiments (as needed for Fig. 1b), (c) the radiomic progression experiment (as needed for Fig. 1c), (d) the radiomic and clinical progression experiment (as needed for Fig. 1d), and (b) the analysis performed for ML model interpretation using the results from (a) and (c). The "Bootstrapped Resampling Machine Learning Experiment" process blocks in (a), (b), (d) each represent an instance of the ML experiment common template described in the article text under heading "Machine learning experimental design" and shown schematically above in Fig. S1 of this supplementary document. The specific model inputs/outputs and experimental results associated with each experiment instance are shown in (a), (b), and (d), on which further analysis is performed. From each ML experiment instance, there are training and testing dataset prediction probabilities per bootstrapped resampling iteration. To get the "Average Prediction Probability (Per BM)", the testing dataset probabilities are iterated through to find all instances in which a given BM was randomly chosen to be in the testing dataset for that bootstrapped iteration. The prediction probabilities for the BM from all these instances are then aggregated and their average taken. This process is then repeated for each BM in the entire dataset. A similar process is performed on the "Training Dataset Prediction Probabilities" when the "Choose…" process blocks are used, except in these cases the training dataset probabilities are just aggregated according to the "Choose…" process rule, with no average taken, allowing for the creation of an average ROC from the chosen training dataset probabilities instead. The specific details of the model interpretation analysis processes outlined in (b) are provided in the article text under the heading "Post-SRS progression machine learning model interpretation".

		Expert 1					Expert 2					Expert 3					Trainee 1					Trainee 2				
		A	B	C	D	E	A	B	C	D	E	A	B	C	D	E	A	B	C	D	E	A	B	C	D	E
Expert 1	A	**40**					**19**	19	2	0	0	**14**	19	2	2	3	**16**	22	0	0	2	**11**	20	4	2	3
Expert 1	B		**27**				1	**23**	0	1	2	0	**10**	1	2	14	0	**9**	1	3	14	0	**19**	2	1	5
Expert 1	C			**27**			1	7	**9**	7	3	2	3	**7**	10	5	1	7	**11**	2	6	0	3	**17**	4	3
Expert 1	D				**14**		0	2	0	**7**	5	0	0	0	**3**	11	0	2	0	**9**	3	0	2	0	**7**	5
Expert 1	E					**15**	0	8	1	2	**4**	0	3	2	4	**6**	0	5	0	2	**8**	0	6	3	0	**6**
Expert 2	A						**21**					**14**	6	0	0	1	**14**	5	0	0	2	**9**	7	1	1	3
Expert 2	B							**59**				2	**29**	3	6	19	3	**31**	1	3	21	2	**41**	8	3	5
Expert 2	C								**12**			0	0	**9**	3	0	0	7	**5**	0	0	0	0	**12**	0	0
Expert 2	D									**17**		0	0	0	**11**	6	0	0	5	**11**	1	0	2	4	**8**	3
Expert 2	E										**14**	0	0	0	1	**13**	0	2	1	2	**9**	0	0	1	2	**11**
Expert 3	A											**16**					**11**	3	0	0	2	**9**	3	1	1	2
Expert 3	B												**35**				4	**23**	0	2	6	2	**29**	4	0	0
Expert 3	C													**12**			0	7	**4**	0	1	0	0	**12**	0	0
Expert 3	D														**21**		1	3	5	**6**	6	0	4	9	**7**	1
Expert 3	E															**39**	1	9	3	8	**18**	0	14	0	6	**19**
Trainee 1	A																**17**					**7**	4	2	2	2
Trainee 1	B																	**45**				4	**27**	11	1	2
Trainee 1	C																		**12**			0	0	**9**	1	2
Trainee 1	D																			**16**		0	5	0	**7**	4
Trainee 1	E																				**33**	0	14	4	3	**12**
Trainee 2	A																					**11**				
Trainee 2	B																						**50**			
Trainee 2	C																							**26**		
Trainee 2	D																								**14**	
Trainee 2	E																									**22**

Appearance Legend:

A: Homogeneous
B: Heterogeneous
C: Cystic (Simple)
D: Cystic (Complex)
E: Necrotic

Table S5. Confusion matrices across appearance labels (A–E), for each pairwise comparison between observers. The highlighted cells indicate instances of agreement between observers, allowing for the calculation of the agreement rate when summed and divided by the total number of BMs (*n* = 123). The diagonal of the larger "observer-level" matrix only contains these highlighted cells as each observer is in perfect agreement with themselves, and so these values provide the number of each appearance label an observer called.

(a) **Qualitative Appearance**

	A	B	C	D	E
A	0.0%	21.4%	2.7%	1.5%	3.6%
B		0.0%	11.1%	7.1%	25.3%
C			0.0%	8.5%	6.0%
D				0.0%	12.8%
E					0.0%

Qualitative Appearance

Appearance Legend:
A: Homogeneous
B: Heterogeneous
C: Cystic (Simple)
D: Cystic (Complex)
E: Necrotic

(b) **Qualitative Appearance**

	A	B	C	D	E
A	0.0%	24.6%	3.7%	1.0%	2.1%
B		0.0%	7.3%	5.8%	24.1%
C			0.0%	10.5%	5.8%
D				0.0%	15.2%
E					0.0%

Qualitative Appearance

Appearance Legend:
A: Homogeneous
B: Heterogeneous
C: Cystic (Simple)
D: Cystic (Complex)
E: Necrotic

Table S6. Percentage of disagreements arising across observer pairs based upon qualitative appearances from each observer. (a) shows disagreement percentages from all observers, while (b) is only from expert observers. A given value in a table (e.g. column “B”, row “A”) represents how many disagreements across all observer pairs occurred when one observer selected an certain appearance (e.g. “B” or “Heterogeneous”) and the other observer selected a certain alternate appearance (e.g. “A” or Homogeneous).

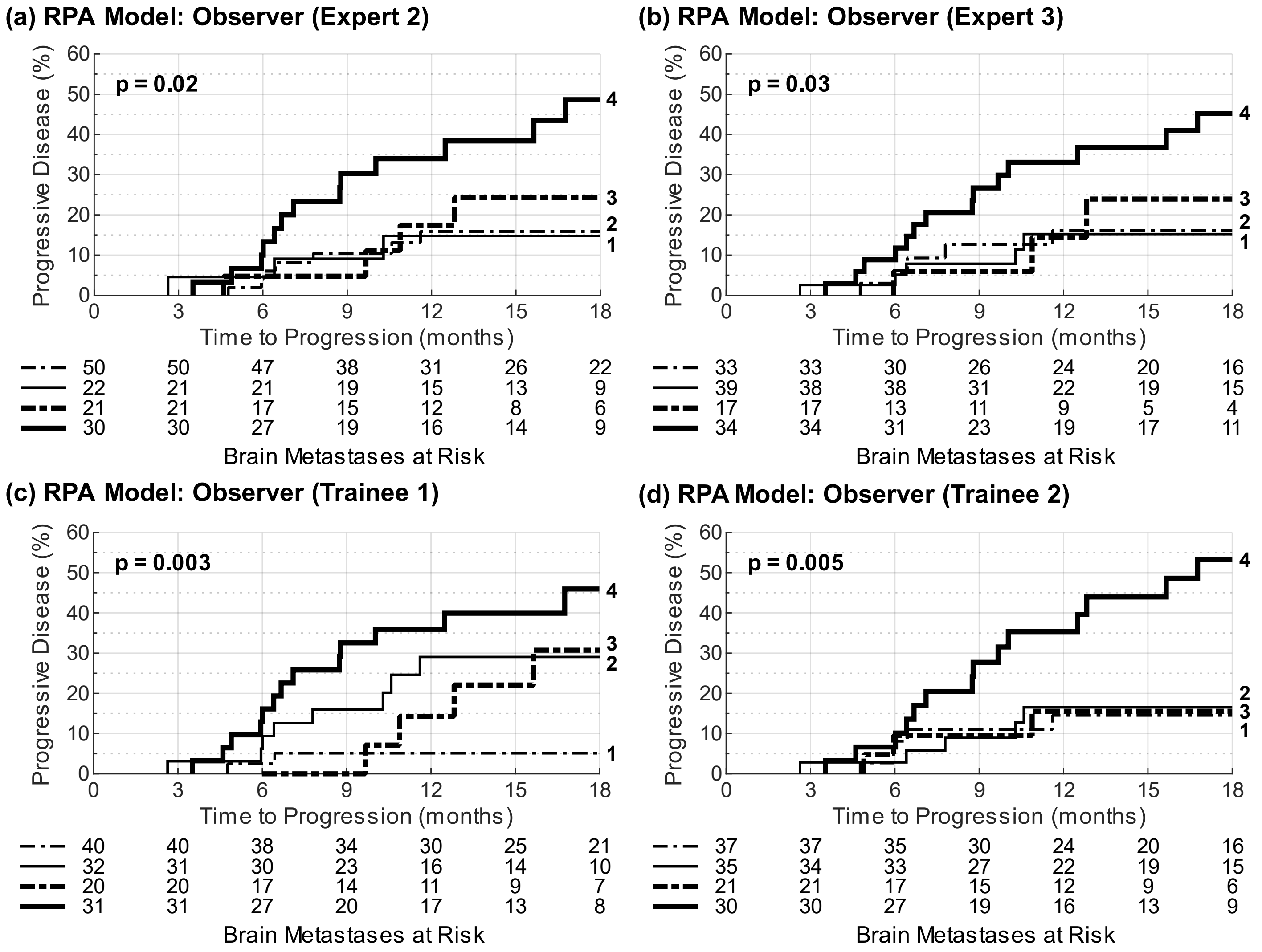

Figure S3. KM analysis plots for progressive disease for each individual observer for comparison against Expert 1 (see Fig. 2a). The risk group number for each risk curve is labelled on the right y-axis, and the number of BMs at risk per 3-month follow-up interval is given below each x-axis. The stated *p*-values are from the log-rank test performed over all risk groups.

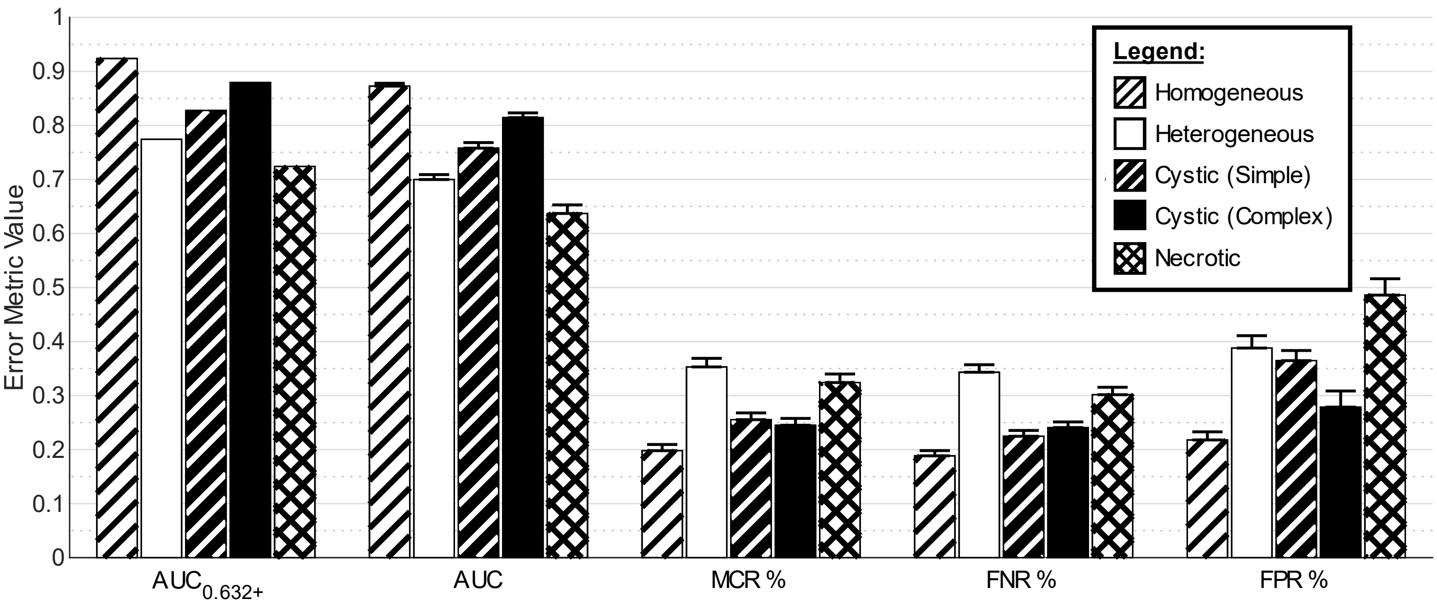

Figure S4: Error metrics from the radiomic Expert 1 appearance experiments using the observer labels from the original study. As each appearance label (e.g. “homogeneous”) had specific models trained to make a binary labelling decision (e.g. “homogeneous” or “not homogeneous”), error metrics for each appearance label are presented. The error bars for the non-$AUC_{0.632+}$ error metrics represent the 95% confidence interval of each value determined from the 250 bootstrapped resampling iterations.

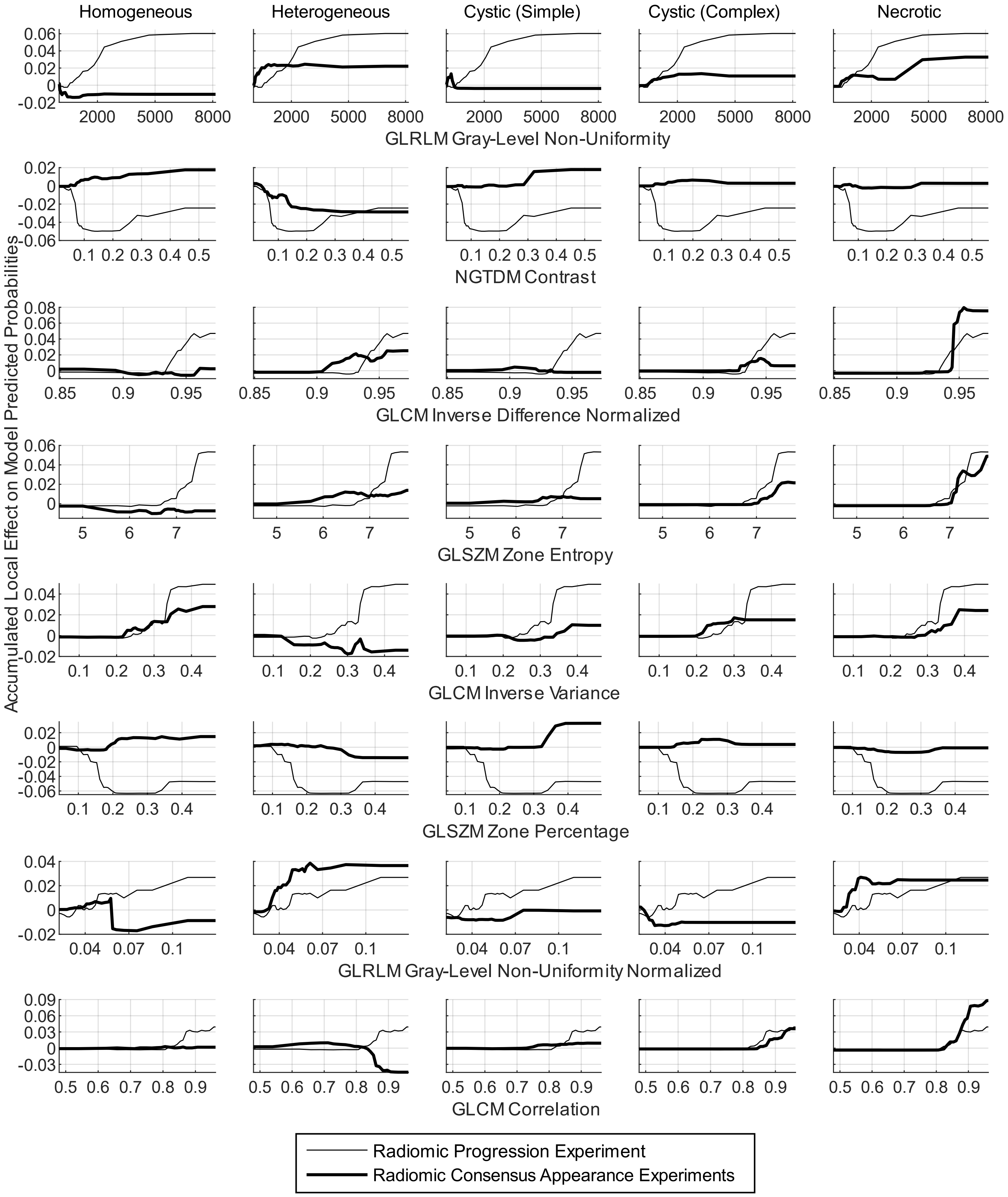

Figure S5. ALE plots for the highly importance features from Table 3 that were not included in Fig. 5, as these features were not also highly important for any of the radiomic appearance label experiments. The Pearson correlation coefficient values for each plot's pair of ALE curve are given in Table 3.
